# Supplementary material for: Poorly Expressed Alleles of Several Human Immunoglobulin Heavy Chain Variable Genes are Common in the Human Population
Source: Front Immunol. 2021 Feb 24;11:603980. doi: 10.3389/fimmu.2020.603980 (PMC7943739; doi:10.3389/fimmu.2020.603980)
Supplement: Supplementary Figure 2 — Visualization of expression levels, as inferred by IgDiscover technology (8), of IGHV genes found in 35 genotypes of subjects for which haplotyping, based on heterozygosity of IGHJ6, is possible. [file Image_2.pdf]

Supplementary Figure 2. Visualization of expression levels, as inferred by IgDiscover technology (Corcoran et al., 2016), of IGHV genes found in 35 genotypes of subjects for which haplotyping, based on heterozygosity of IGHJ6, is possible.

ERR2567187

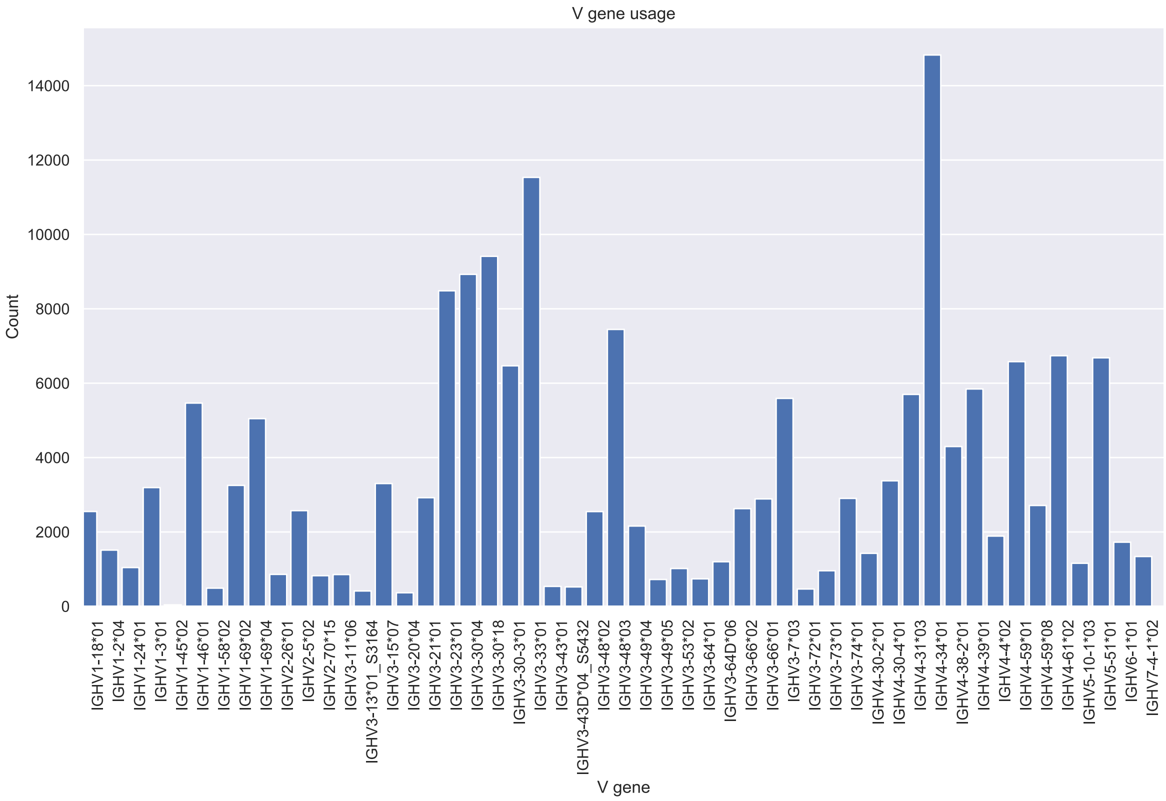
V gene usage
Count
14000
12000
10000
8000
6000
4000
2000
0
IGHV1-18*01
IGHV1-2*04
IGHV1-24*01
IGHV1-3*01
IGHV1-45*02
IGHV1-46*01
IGHV1-58*02
IGHV1-69*02
IGHV1-69*04
IGHV2-26*01
IGHV2-5*02
IGHV2-70*15
IGHV3-11*06
IGHV3-13*01_S3164
IGHV3-15*07
IGHV3-20*04
IGHV3-21*01
IGHV3-23*01
IGHV3-30*04
IGHV3-30*18
IGHV3-30-3*01
IGHV3-33*01
IGHV3-43*01
IGHV3-43D*04_S5432
IGHV3-48*02
IGHV3-48*03
IGHV3-49*04
IGHV3-49*05
IGHV3-53*02
IGHV3-64*01
IGHV3-64D*06
IGHV3-66*02
IGHV3-66*01
IGHV3-7*03
IGHV3-72*01
IGHV3-73*01
IGHV3-74*01
IGHV4-30-2*01
IGHV4-30-4*01
IGHV4-31*03
IGHV4-34*01
IGHV4-38-2*01
IGHV4-39*01
IGHV4-4*02
IGHV4-59*01
IGHV4-59*08
IGHV4-61*02
IGHV5-10-1*03
IGHV5-51*01
IGHV6-1*01
IGHV7-4-1*02
V gene

ERR2567189

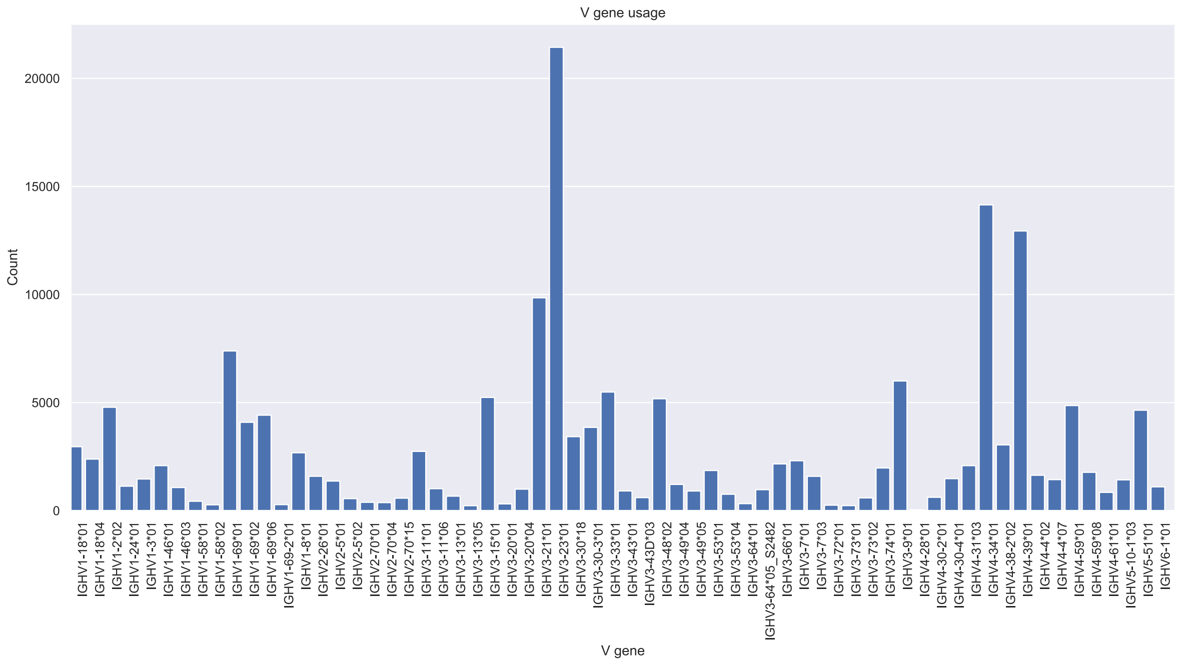
V gene usage
Count
20000
15000
10000
5000
0
IGHV1-18*01
IGHV1-18*04
IGHV1-2*02
IGHV1-24*01
IGHV1-3*01
IGHV1-46*01
IGHV1-46*03
IGHV1-58*01
IGHV1-58*02
IGHV1-69*01
IGHV1-69*02
IGHV1-69*06
IGHV1-69-2*01
IGHV1-8*01
IGHV2-26*01
IGHV2-5*01
IGHV2-5*02
IGHV2-70*01
IGHV2-70*04
IGHV2-70*15
IGHV3-11*01
IGHV3-11*06
IGHV3-13*01
IGHV3-13*05
IGHV3-15*01
IGHV3-20*01
IGHV3-20*04
IGHV3-21*01
IGHV3-23*01
IGHV3-30*18
IGHV3-30-3*01
IGHV3-33*01
IGHV3-43*01
IGHV3-43D*03
IGHV3-48*02
IGHV3-49*04
IGHV3-49*05
IGHV3-53*01
IGHV3-53*04
IGHV3-64*01
IGHV3-64*05_S2482
IGHV3-66*01
IGHV3-7*01
IGHV3-7*03
IGHV3-72*01
IGHV3-73*01
IGHV3-73*02
IGHV3-74*01
IGHV3-9*01
IGHV4-28*01
IGHV4-30-2*01
IGHV4-30-4*01
IGHV4-31*03
IGHV4-34*01
IGHV4-38-2*02
IGHV4-39*01
IGHV4-4*02
IGHV4-4*07
IGHV4-59*01
IGHV4-59*08
IGHV4-61*01
IGHV5-10-1*03
IGHV5-51*01
IGHV6-1*01
V gene

ERR2567192

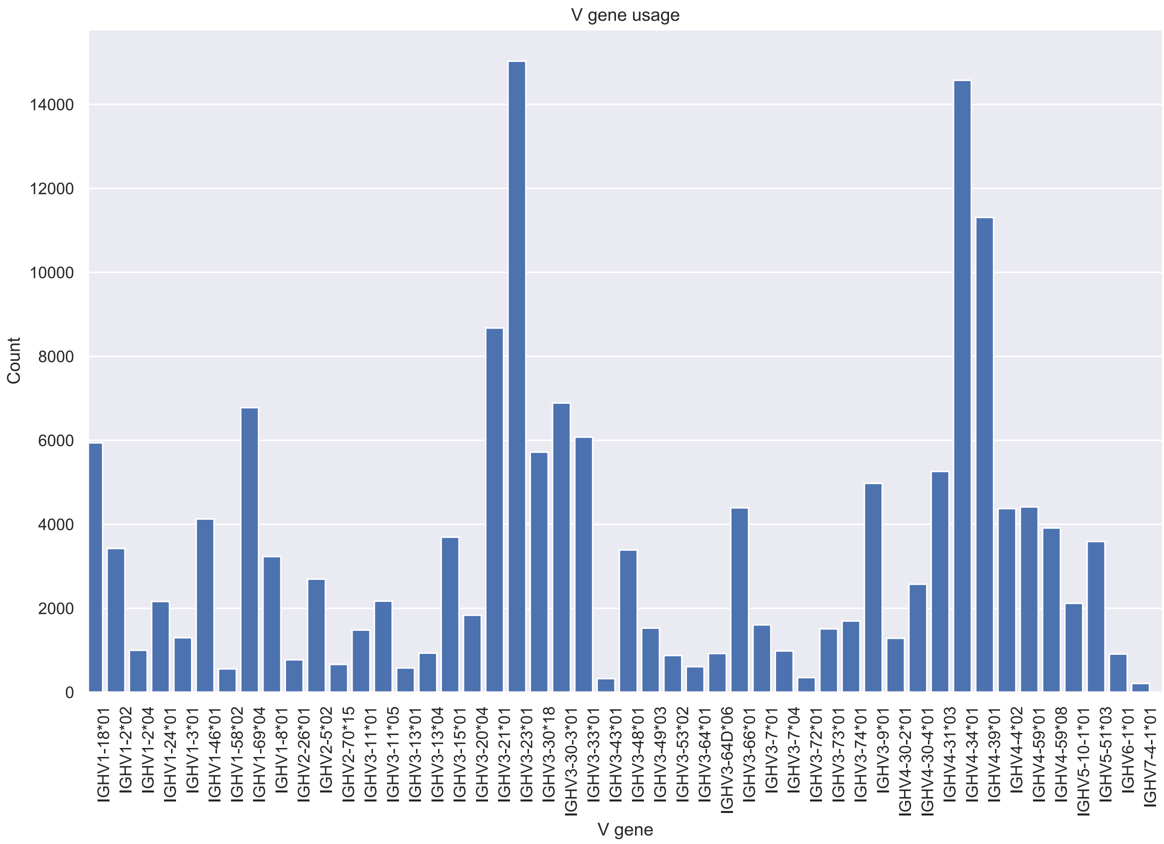
V gene usage
Count
0
2000
4000
6000
8000
10000
12000
14000
IGHV1-18*01
IGHV1-2*02
IGHV1-2*04
IGHV1-24*01
IGHV1-3*01
IGHV1-46*01
IGHV1-58*02
IGHV1-69*04
IGHV1-8*01
IGHV2-26*01
IGHV2-5*02
IGHV2-70*15
IGHV3-11*01
IGHV3-11*05
IGHV3-13*01
IGHV3-13*04
IGHV3-15*01
IGHV3-20*04
IGHV3-21*01
IGHV3-23*01
IGHV3-30*18
IGHV3-30-3*01
IGHV3-33*01
IGHV3-43*01
IGHV3-48*01
IGHV3-49*03
IGHV3-53*02
IGHV3-64*01
IGHV3-64D*06
IGHV3-66*01
IGHV3-7*01
IGHV3-7*04
IGHV3-72*01
IGHV3-73*01
IGHV3-74*01
IGHV3-9*01
IGHV4-30-2*01
IGHV4-30-4*01
IGHV4-31*03
IGHV4-34*01
IGHV4-39*01
IGHV4-4*02
IGHV4-59*01
IGHV4-59*08
IGHV5-10-1*01
IGHV5-51*03
IGHV6-1*01
IGHV7-4-1*01
V gene

ERR2567199

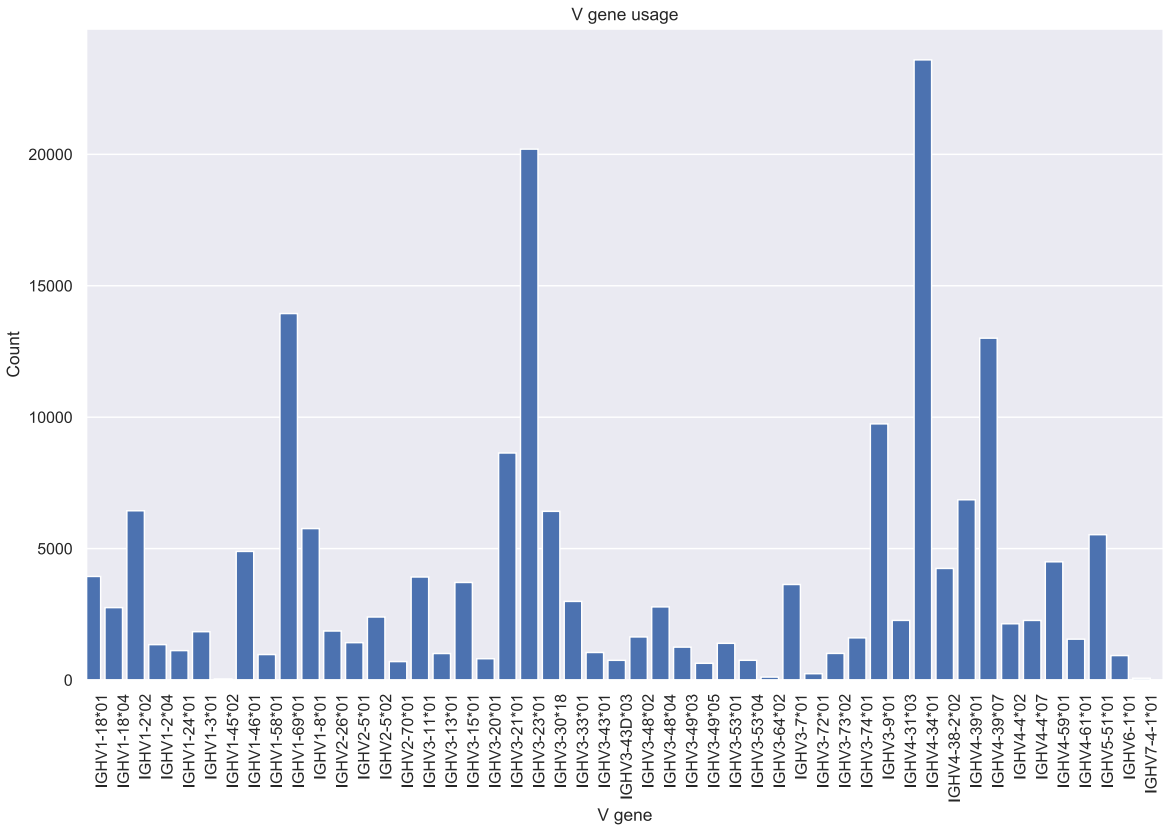
V gene usage
Count
0
5000
10000
15000
20000
V gene
IGHV1-18*01
IGHV1-18*04
IGHV1-2*02
IGHV1-2*04
IGHV1-24*01
IGHV1-3*01
IGHV1-45*02
IGHV1-46*01
IGHV1-58*01
IGHV1-69*01
IGHV1-8*01
IGHV2-26*01
IGHV2-5*01
IGHV2-5*02
IGHV2-70*01
IGHV3-11*01
IGHV3-13*01
IGHV3-15*01
IGHV3-20*01
IGHV3-21*01
IGHV3-23*01
IGHV3-30*18
IGHV3-33*01
IGHV3-43*01
IGHV3-43D*03
IGHV3-48*02
IGHV3-48*04
IGHV3-49*03
IGHV3-49*05
IGHV3-53*01
IGHV3-53*04
IGHV3-64*02
IGHV3-7*01
IGHV3-72*01
IGHV3-73*02
IGHV3-74*01
IGHV3-9*01
IGHV4-31*03
IGHV4-34*01
IGHV4-38-2*02
IGHV4-39*01
IGHV4-39*07
IGHV4-4*02
IGHV4-4*07
IGHV4-59*01
IGHV4-61*01
IGHV5-51*01
IGHV6-1*01
IGHV7-4-1*01

ERR2567200

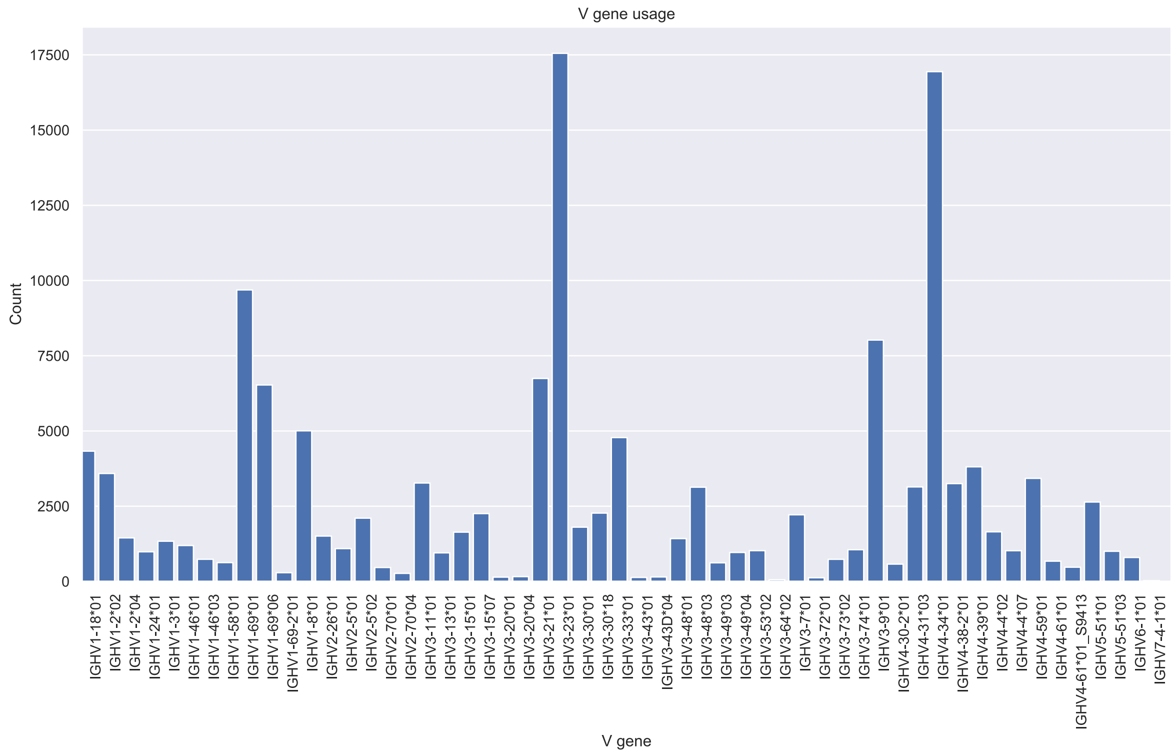
V gene usage
Count
17500
15000
12500
10000
7500
5000
2500
0
IGHV1-18*01
IGHV1-2*02
IGHV1-2*04
IGHV1-24*01
IGHV1-3*01
IGHV1-46*01
IGHV1-46*03
IGHV1-58*01
IGHV1-69*01
IGHV1-69*06
IGHV1-69-2*01
IGHV1-8*01
IGHV2-26*01
IGHV2-5*01
IGHV2-5*02
IGHV2-70*01
IGHV2-70*04
IGHV3-11*01
IGHV3-13*01
IGHV3-15*01
IGHV3-15*07
IGHV3-20*01
IGHV3-20*04
IGHV3-21*01
IGHV3-23*01
IGHV3-30*01
IGHV3-30*18
IGHV3-33*01
IGHV3-43*01
IGHV3-43D*04
IGHV3-48*01
IGHV3-48*03
IGHV3-49*03
IGHV3-49*04
IGHV3-53*02
IGHV3-64*02
IGHV3-7*01
IGHV3-72*01
IGHV3-73*02
IGHV3-74*01
IGHV3-9*01
IGHV4-30-2*01
IGHV4-31*03
IGHV4-34*01
IGHV4-38-2*01
IGHV4-39*01
IGHV4-4*02
IGHV4-4*07
IGHV4-59*01
IGHV4-61*01
IGHV4-61*01_S9413
IGHV5-51*01
IGHV5-51*03
IGHV6-1*01
IGHV7-4-1*01
V gene

ERR2567201

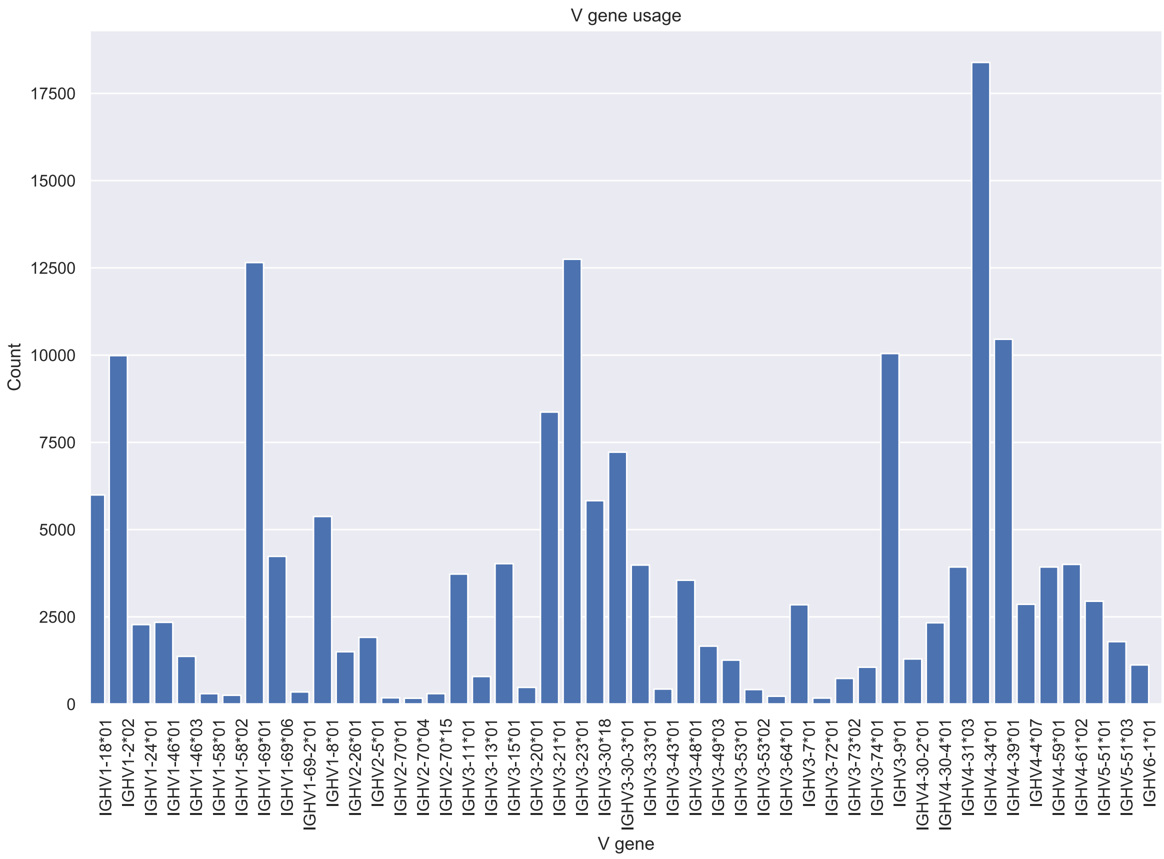
V gene usage
Count
V gene
0
2500
5000
7500
10000
12500
15000
17500
IGHV1-18*01
IGHV1-2*02
IGHV1-24*01
IGHV1-46*01
IGHV1-46*03
IGHV1-58*01
IGHV1-58*02
IGHV1-69*01
IGHV1-69*06
IGHV1-69-2*01
IGHV1-8*01
IGHV2-26*01
IGHV2-5*01
IGHV2-70*01
IGHV2-70*04
IGHV2-70*15
IGHV3-11*01
IGHV3-13*01
IGHV3-15*01
IGHV3-20*01
IGHV3-21*01
IGHV3-23*01
IGHV3-30*18
IGHV3-30-3*01
IGHV3-33*01
IGHV3-43*01
IGHV3-48*01
IGHV3-49*03
IGHV3-53*01
IGHV3-53*02
IGHV3-64*01
IGHV3-7*01
IGHV3-72*01
IGHV3-73*02
IGHV3-74*01
IGHV3-9*01
IGHV4-30-2*01
IGHV4-30-4*01
IGHV4-31*03
IGHV4-34*01
IGHV4-39*01
IGHV4-4*07
IGHV4-59*01
IGHV4-61*02
IGHV5-51*01
IGHV5-51*03
IGHV6-1*01

ERR2567204

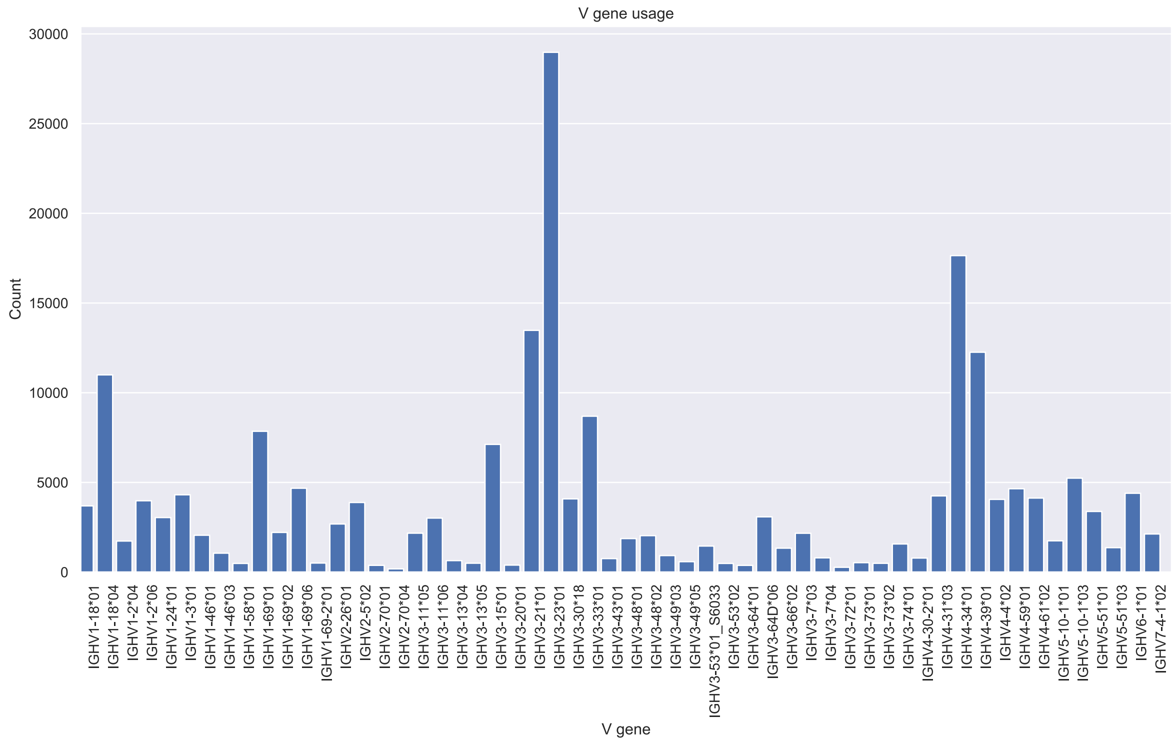
V gene usage
30000
25000
20000
15000
10000
5000
0
Count
IGHV1-18*01
IGHV1-18*04
IGHV1-2*04
IGHV1-2*06
IGHV1-24*01
IGHV1-3*01
IGHV1-46*01
IGHV1-46*03
IGHV1-58*01
IGHV1-69*01
IGHV1-69*02
IGHV1-69*06
IGHV1-69-2*01
IGHV2-26*01
IGHV2-5*02
IGHV2-70*01
IGHV2-70*04
IGHV3-11*05
IGHV3-11*06
IGHV3-13*04
IGHV3-13*05
IGHV3-15*01
IGHV3-20*01
IGHV3-21*01
IGHV3-23*01
IGHV3-30*18
IGHV3-33*01
IGHV3-43*01
IGHV3-48*01
IGHV3-48*02
IGHV3-49*03
IGHV3-49*05
IGHV3-53*01_S6033
IGHV3-53*02
IGHV3-64*01
IGHV3-64D*06
IGHV3-66*02
IGHV3-7*03
IGHV3-7*04
IGHV3-72*01
IGHV3-73*01
IGHV3-73*02
IGHV3-74*01
IGHV4-30-2*01
IGHV4-31*03
IGHV4-34*01
IGHV4-39*01
IGHV4-4*02
IGHV4-59*01
IGHV4-61*02
IGHV5-10-1*01
IGHV5-10-1*03
IGHV5-51*01
IGHV5-51*03
IGHV6-1*01
IGHV7-4-1*02
V gene

ERR2567206

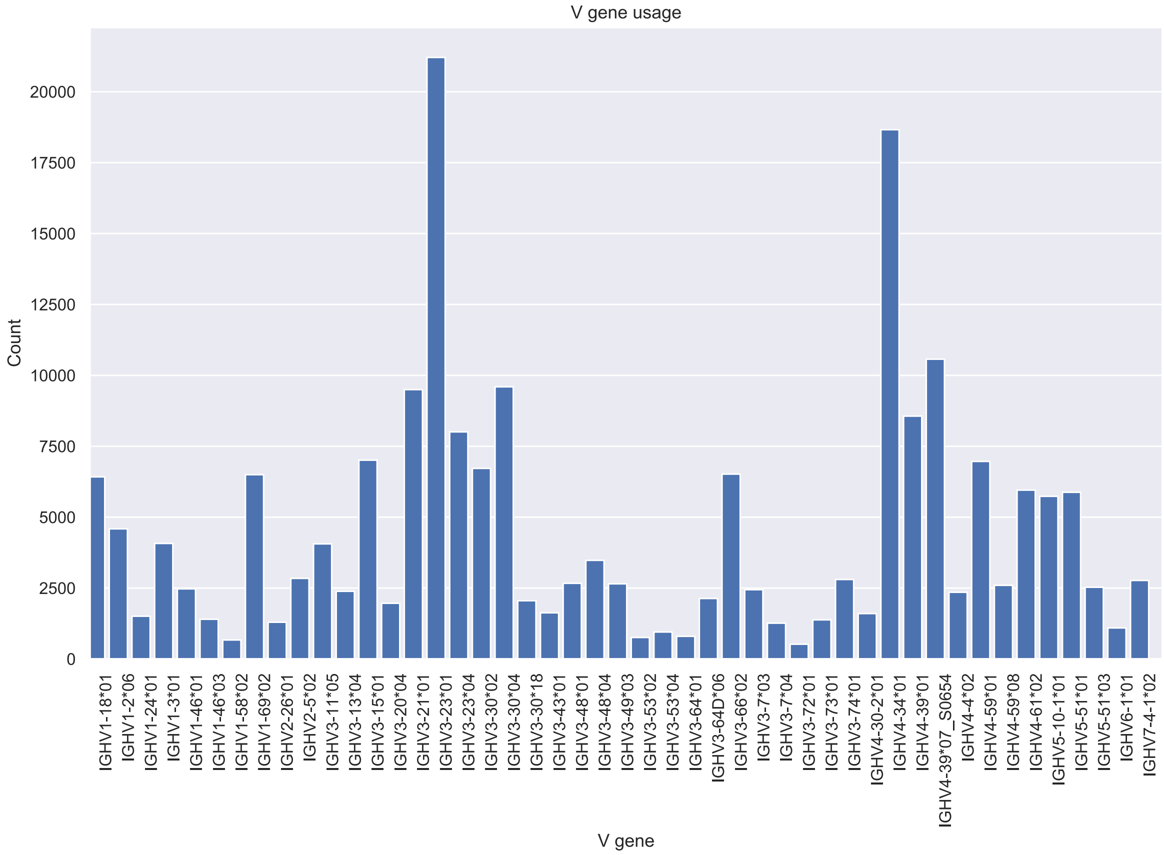

V gene usage
Count
0
2500
5000
7500
10000
12500
15000
17500
20000
V gene
IGHV1-18*01
IGHV1-2*06
IGHV1-24*01
IGHV1-3*01
IGHV1-46*01
IGHV1-46*03
IGHV1-58*02
IGHV1-69*02
IGHV2-26*01
IGHV2-5*02
IGHV3-11*05
IGHV3-13*04
IGHV3-15*01
IGHV3-20*04
IGHV3-21*01
IGHV3-23*01
IGHV3-23*04
IGHV3-30*02
IGHV3-30*04
IGHV3-30*18
IGHV3-43*01
IGHV3-48*01
IGHV3-48*04
IGHV3-49*03
IGHV3-53*02
IGHV3-53*04
IGHV3-64*01
IGHV3-64D*06
IGHV3-66*02
IGHV3-7*03
IGHV3-7*04
IGHV3-72*01
IGHV3-73*01
IGHV3-74*01
IGHV4-30-2*01
IGHV4-34*01
IGHV4-39*01
IGHV4-39*07_S0654
IGHV4-4*02
IGHV4-59*01
IGHV4-59*08
IGHV4-61*02
IGHV5-10-1*01
IGHV5-51*01
IGHV5-51*03
IGHV6-1*01
IGHV7-4-1*02

ERR2567213

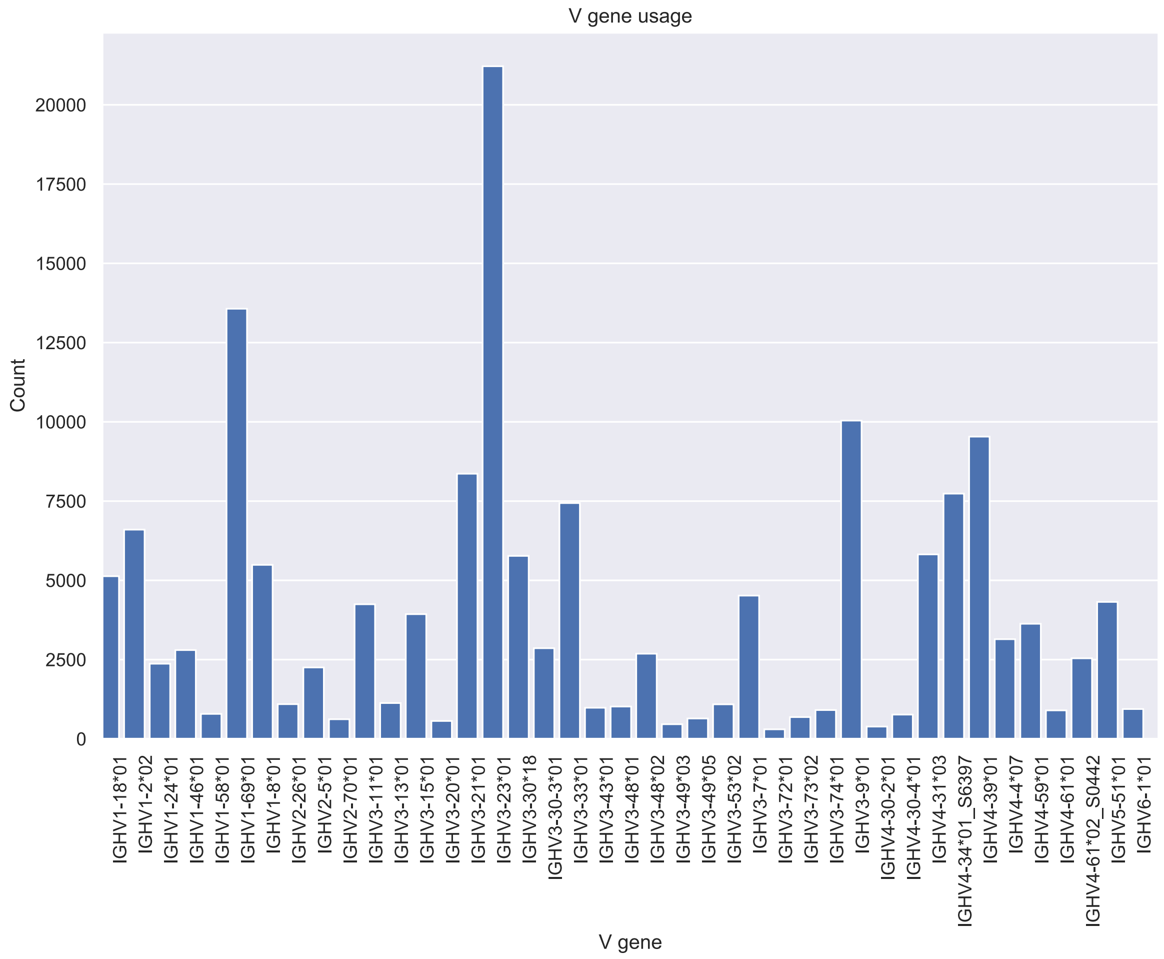
V gene usage
Count
V gene
0
2500
5000
7500
10000
12500
15000
17500
20000
IGHV1-18*01
IGHV1-2*02
IGHV1-24*01
IGHV1-46*01
IGHV1-58*01
IGHV1-69*01
IGHV1-8*01
IGHV2-26*01
IGHV2-5*01
IGHV2-70*01
IGHV3-11*01
IGHV3-13*01
IGHV3-15*01
IGHV3-20*01
IGHV3-21*01
IGHV3-23*01
IGHV3-30*18
IGHV3-30-3*01
IGHV3-33*01
IGHV3-43*01
IGHV3-48*01
IGHV3-48*02
IGHV3-49*03
IGHV3-49*05
IGHV3-53*02
IGHV3-7*01
IGHV3-72*01
IGHV3-73*02
IGHV3-74*01
IGHV3-9*01
IGHV4-30-2*01
IGHV4-30-4*01
IGHV4-31*03
IGHV4-34*01_S6397
IGHV4-39*01
IGHV4-4*07
IGHV4-59*01
IGHV4-61*01
IGHV4-61*02_S0442
IGHV5-51*01
IGHV6-1*01

ERR2567214

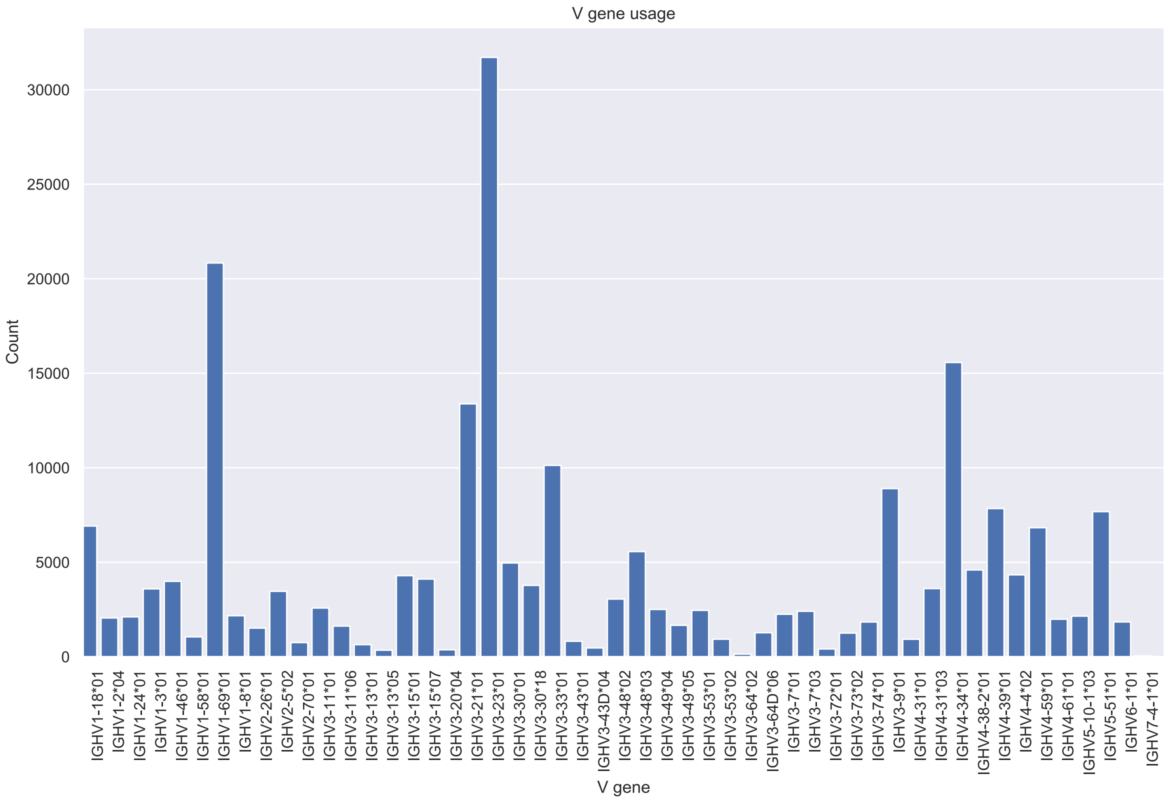

V gene usage
Count
30000
25000
20000
15000
10000
5000
0
IGHV1-18*01
IGHV1-2*04
IGHV1-24*01
IGHV1-3*01
IGHV1-46*01
IGHV1-58*01
IGHV1-69*01
IGHV1-8*01
IGHV2-26*01
IGHV2-5*02
IGHV2-70*01
IGHV3-11*01
IGHV3-11*06
IGHV3-13*01
IGHV3-13*05
IGHV3-15*01
IGHV3-15*07
IGHV3-20*04
IGHV3-21*01
IGHV3-23*01
IGHV3-30*01
IGHV3-30*18
IGHV3-33*01
IGHV3-43*01
IGHV3-43D*04
IGHV3-48*02
IGHV3-48*03
IGHV3-49*04
IGHV3-49*05
IGHV3-53*01
IGHV3-53*02
IGHV3-64*02
IGHV3-64D*06
IGHV3-7*01
IGHV3-7*03
IGHV3-72*01
IGHV3-73*02
IGHV3-74*01
IGHV3-9*01
IGHV4-31*01
IGHV4-31*03
IGHV4-34*01
IGHV4-38-2*01
IGHV4-39*01
IGHV4-4*02
IGHV4-59*01
IGHV4-61*01
IGHV5-10-1*03
IGHV5-51*01
IGHV6-1*01
IGHV7-4-1*01
V gene

ERR2567215

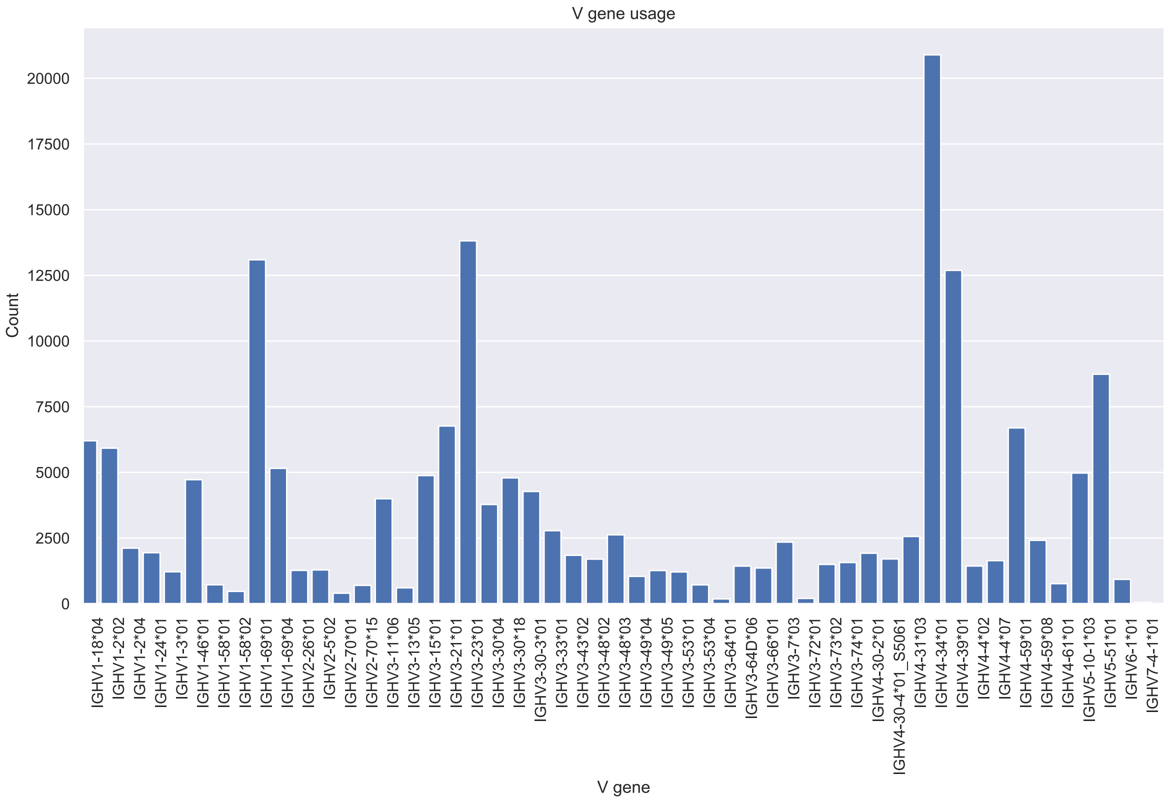
V gene usage
Count
20000
17500
15000
12500
10000
7500
5000
2500
0
IGHV1-18*04
IGHV1-2*02
IGHV1-2*04
IGHV1-24*01
IGHV1-3*01
IGHV1-46*01
IGHV1-58*01
IGHV1-58*02
IGHV1-69*01
IGHV1-69*04
IGHV2-26*01
IGHV2-5*02
IGHV2-70*01
IGHV2-70*15
IGHV3-11*06
IGHV3-13*05
IGHV3-15*01
IGHV3-21*01
IGHV3-23*01
IGHV3-30*04
IGHV3-30*18
IGHV3-30-3*01
IGHV3-33*01
IGHV3-43*02
IGHV3-48*02
IGHV3-48*03
IGHV3-49*04
IGHV3-49*05
IGHV3-53*01
IGHV3-53*04
IGHV3-64*01
IGHV3-64D*06
IGHV3-66*01
IGHV3-7*03
IGHV3-72*01
IGHV3-73*02
IGHV3-74*01
IGHV4-30-2*01
IGHV4-30-4*01_S5061
IGHV4-31*03
IGHV4-34*01
IGHV4-39*01
IGHV4-4*02
IGHV4-4*07
IGHV4-59*01
IGHV4-59*08
IGHV4-61*01
IGHV5-10-1*03
IGHV5-51*01
IGHV6-1*01
IGHV7-4-1*01
V gene

ERR2567217

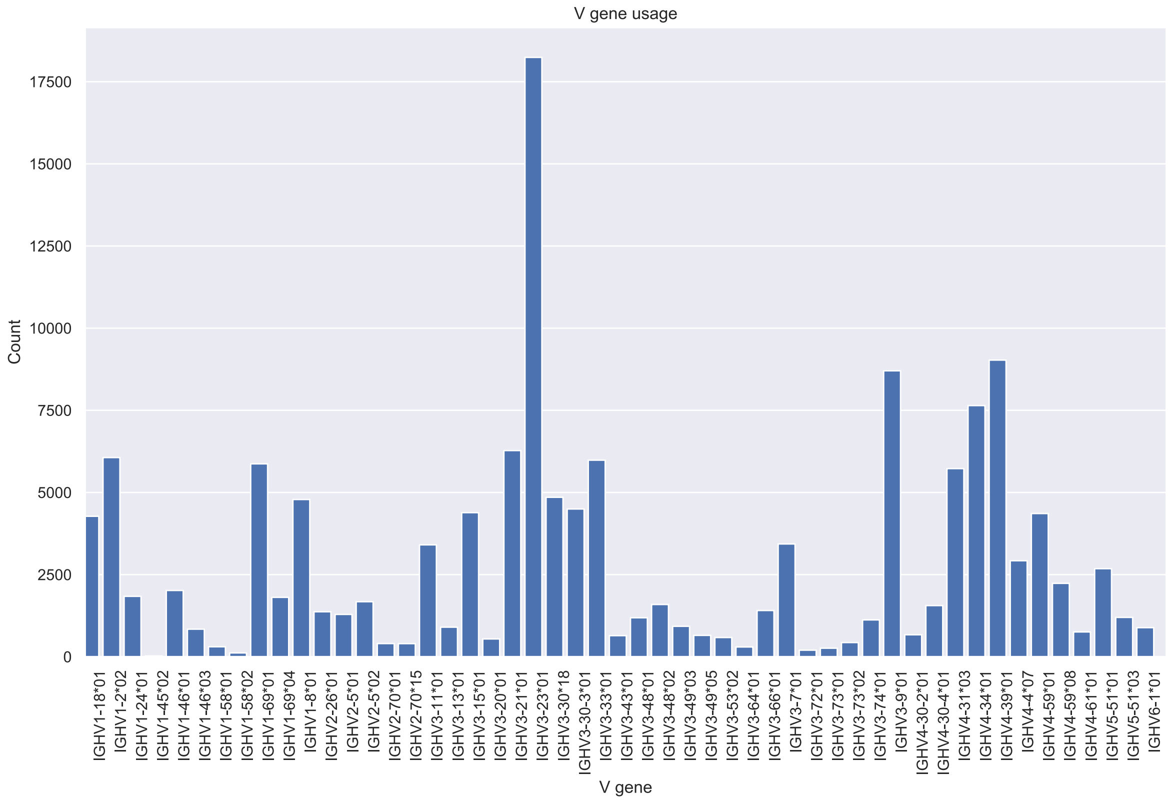
V gene usage
Count
17500
15000
12500
10000
7500
5000
2500
0
IGHV1-18*01
IGHV1-2*02
IGHV1-24*01
IGHV1-45*02
IGHV1-46*01
IGHV1-46*03
IGHV1-58*01
IGHV1-58*02
IGHV1-69*01
IGHV1-69*04
IGHV1-8*01
IGHV2-26*01
IGHV2-5*01
IGHV2-5*02
IGHV2-70*01
IGHV2-70*15
IGHV3-11*01
IGHV3-13*01
IGHV3-15*01
IGHV3-20*01
IGHV3-21*01
IGHV3-23*01
IGHV3-30*18
IGHV3-30-3*01
IGHV3-33*01
IGHV3-43*01
IGHV3-48*01
IGHV3-48*02
IGHV3-49*03
IGHV3-49*05
IGHV3-53*02
IGHV3-64*01
IGHV3-66*01
IGHV3-7*01
IGHV3-72*01
IGHV3-73*01
IGHV3-73*02
IGHV3-74*01
IGHV3-9*01
IGHV4-30-2*01
IGHV4-30-4*01
IGHV4-31*03
IGHV4-34*01
IGHV4-39*01
IGHV4-4*07
IGHV4-59*01
IGHV4-59*08
IGHV4-61*01
IGHV5-51*01
IGHV5-51*03
IGHV6-1*01
V gene

ERR2567220

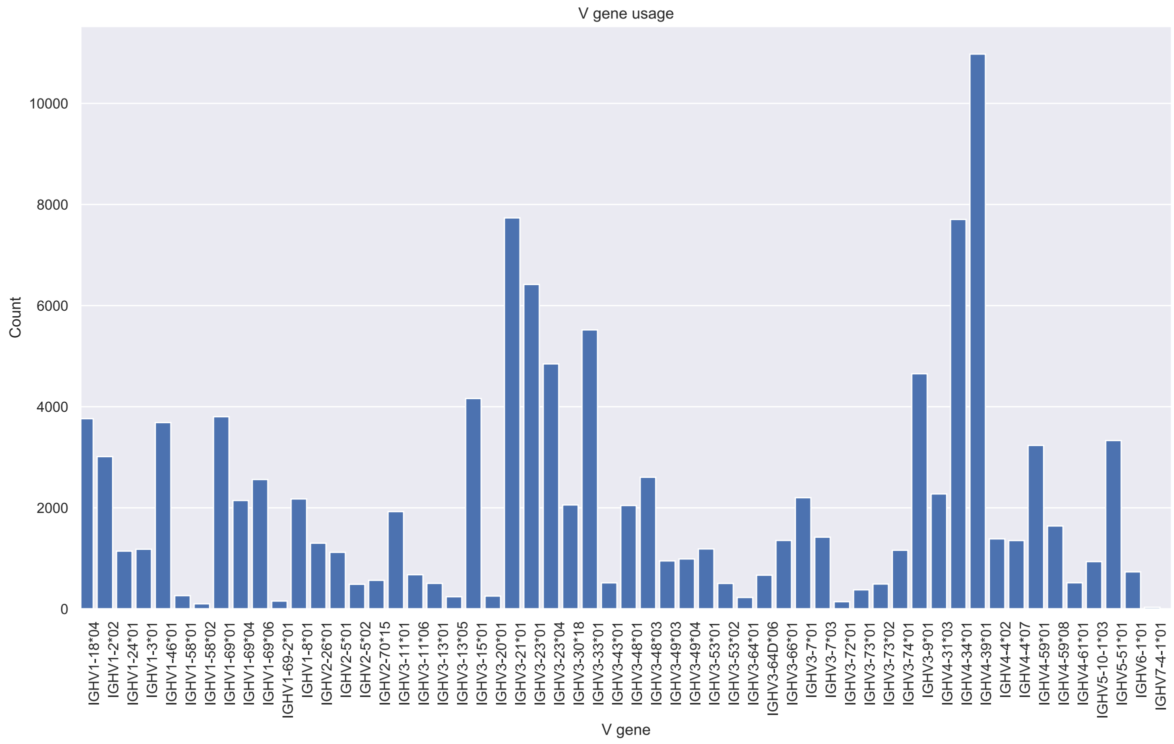
V gene usage
Count
10000
8000
6000
4000
2000
0
IGHV1-18*04
IGHV1-2*02
IGHV1-24*01
IGHV1-3*01
IGHV1-46*01
IGHV1-58*01
IGHV1-58*02
IGHV1-69*01
IGHV1-69*04
IGHV1-69*06
IGHV1-69-2*01
IGHV1-8*01
IGHV2-26*01
IGHV2-5*01
IGHV2-5*02
IGHV2-70*15
IGHV3-11*01
IGHV3-11*06
IGHV3-13*01
IGHV3-13*05
IGHV3-15*01
IGHV3-20*01
IGHV3-21*01
IGHV3-23*01
IGHV3-23*04
IGHV3-30*18
IGHV3-33*01
IGHV3-43*01
IGHV3-48*01
IGHV3-48*03
IGHV3-49*03
IGHV3-49*04
IGHV3-53*01
IGHV3-53*02
IGHV3-64*01
IGHV3-64D*06
IGHV3-66*01
IGHV3-7*01
IGHV3-7*03
IGHV3-72*01
IGHV3-73*01
IGHV3-73*02
IGHV3-74*01
IGHV3-9*01
IGHV4-31*03
IGHV4-34*01
IGHV4-39*01
IGHV4-4*02
IGHV4-4*07
IGHV4-59*01
IGHV4-59*08
IGHV4-61*01
IGHV5-10-1*03
IGHV5-51*01
IGHV6-1*01
IGHV7-4-1*01
V gene

ERR2567221

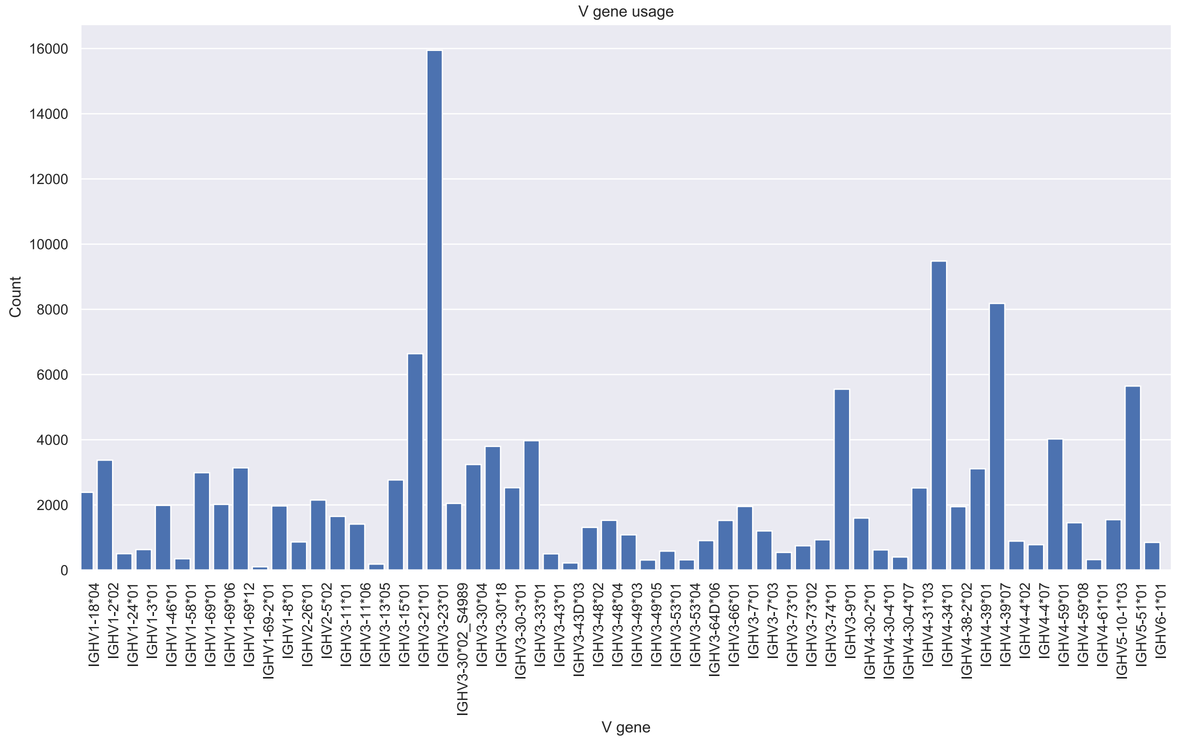
V gene usage
Count
0
2000
4000
6000
8000
10000
12000
14000
16000
V gene
IGHV1-18*04
IGHV1-2*02
IGHV1-24*01
IGHV1-3*01
IGHV1-46*01
IGHV1-58*01
IGHV1-69*01
IGHV1-69*06
IGHV1-69*12
IGHV1-69-2*01
IGHV1-8*01
IGHV2-26*01
IGHV2-5*02
IGHV3-11*01
IGHV3-11*06
IGHV3-13*05
IGHV3-15*01
IGHV3-21*01
IGHV3-23*01
IGHV3-30*02_S4989
IGHV3-30*04
IGHV3-30*18
IGHV3-30-3*01
IGHV3-33*01
IGHV3-43*01
IGHV3-43D*03
IGHV3-48*02
IGHV3-48*04
IGHV3-49*03
IGHV3-49*05
IGHV3-53*01
IGHV3-53*04
IGHV3-64D*06
IGHV3-66*01
IGHV3-7*01
IGHV3-7*03
IGHV3-73*01
IGHV3-73*02
IGHV3-74*01
IGHV3-9*01
IGHV4-30-2*01
IGHV4-30-4*01
IGHV4-30-4*07
IGHV4-31*03
IGHV4-34*01
IGHV4-38-2*02
IGHV4-39*01
IGHV4-39*07
IGHV4-4*02
IGHV4-4*07
IGHV4-59*01
IGHV4-59*08
IGHV4-61*01
IGHV5-10-1*03
IGHV5-51*01
IGHV6-1*01

ERR2567223

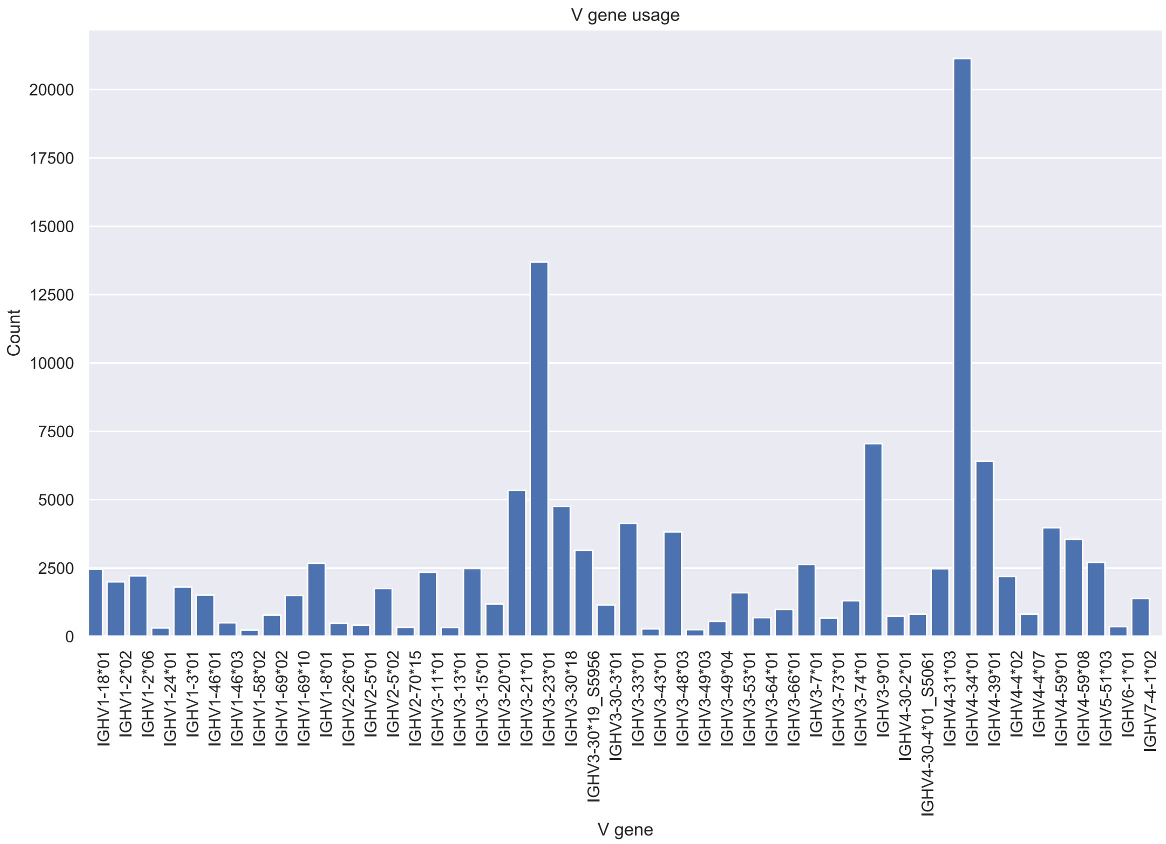

V gene usage
Count
20000
17500
15000
12500
10000
7500
5000
2500
0
IGHV1-18*01
IGHV1-2*02
IGHV1-2*06
IGHV1-24*01
IGHV1-3*01
IGHV1-46*01
IGHV1-46*03
IGHV1-58*02
IGHV1-69*02
IGHV1-69*10
IGHV1-8*01
IGHV2-26*01
IGHV2-5*01
IGHV2-5*02
IGHV2-70*15
IGHV3-11*01
IGHV3-13*01
IGHV3-15*01
IGHV3-20*01
IGHV3-21*01
IGHV3-23*01
IGHV3-30*18
IGHV3-30*19_S5956
IGHV3-30-3*01
IGHV3-33*01
IGHV3-43*01
IGHV3-48*03
IGHV3-49*03
IGHV3-49*04
IGHV3-53*01
IGHV3-64*01
IGHV3-66*01
IGHV3-7*01
IGHV3-73*01
IGHV3-74*01
IGHV3-9*01
IGHV4-30-2*01
IGHV4-30-4*01_S5061
IGHV4-31*03
IGHV4-34*01
IGHV4-39*01
IGHV4-4*02
IGHV4-4*07
IGHV4-59*01
IGHV4-59*08
IGHV5-51*03
IGHV6-1*01
IGHV7-4-1*02
V gene

ERR2567226

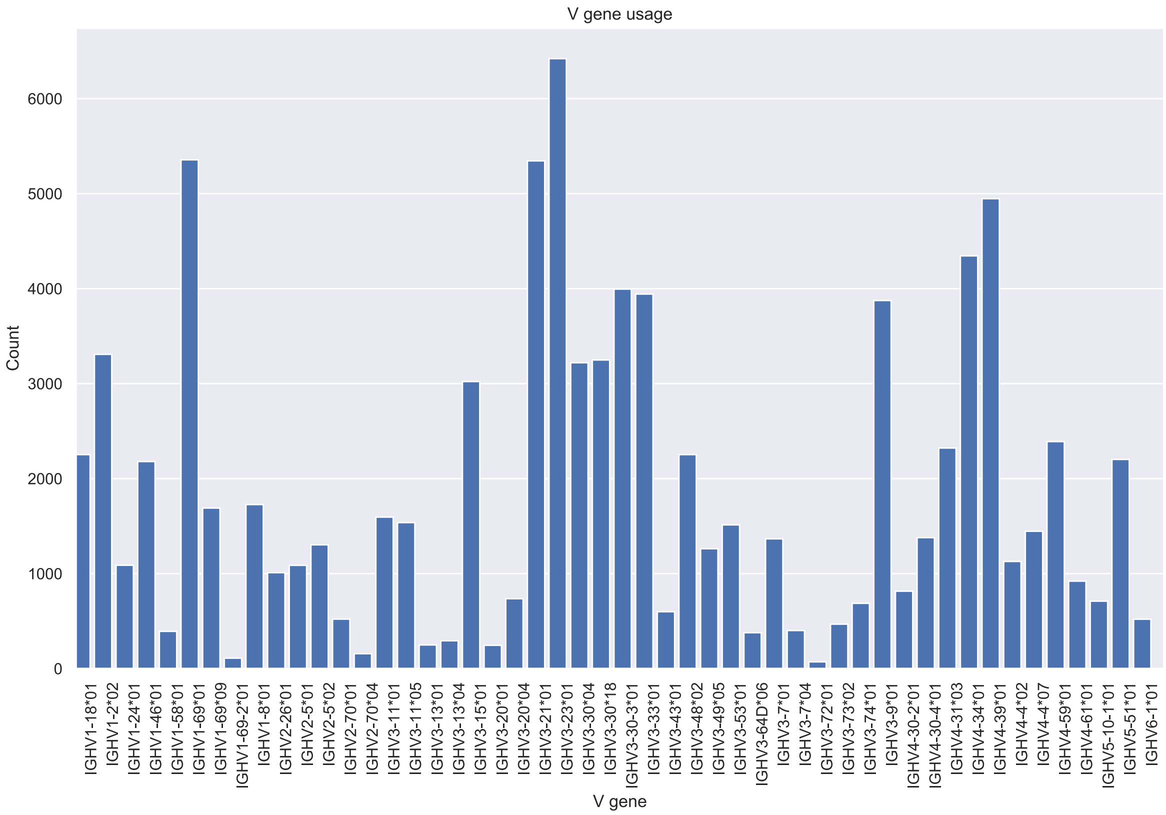

V gene usage
Count
0
1000
2000
3000
4000
5000
6000
V gene
IGHV1-18*01
IGHV1-2*02
IGHV1-24*01
IGHV1-46*01
IGHV1-58*01
IGHV1-69*01
IGHV1-69*09
IGHV1-69-2*01
IGHV1-8*01
IGHV2-26*01
IGHV2-5*01
IGHV2-5*02
IGHV2-70*01
IGHV2-70*04
IGHV3-11*01
IGHV3-11*05
IGHV3-13*01
IGHV3-13*04
IGHV3-15*01
IGHV3-20*01
IGHV3-20*04
IGHV3-21*01
IGHV3-23*01
IGHV3-30*04
IGHV3-30*18
IGHV3-30-3*01
IGHV3-33*01
IGHV3-43*01
IGHV3-48*02
IGHV3-49*05
IGHV3-53*01
IGHV3-64D*06
IGHV3-7*01
IGHV3-7*04
IGHV3-72*01
IGHV3-73*02
IGHV3-74*01
IGHV3-9*01
IGHV4-30-2*01
IGHV4-30-4*01
IGHV4-31*03
IGHV4-34*01
IGHV4-39*01
IGHV4-4*02
IGHV4-4*07
IGHV4-59*01
IGHV4-61*01
IGHV5-10-1*01
IGHV5-51*01
IGHV6-1*01

ERR2567230

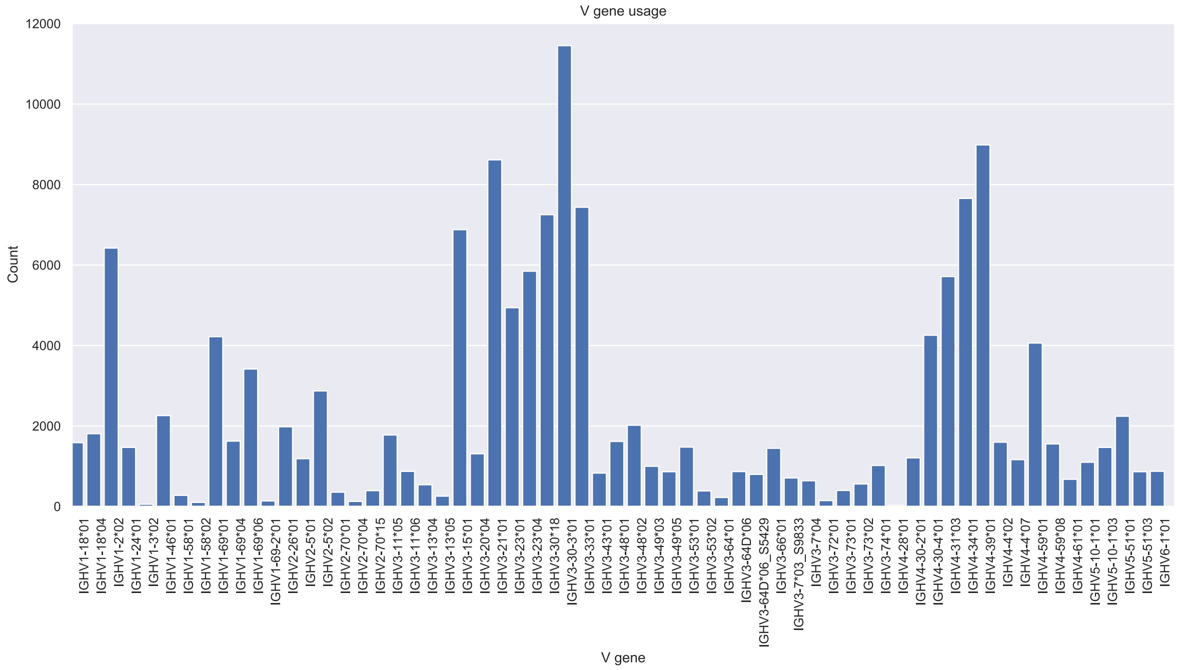
V gene usage
Count
12000
10000
8000
6000
4000
2000
0
IGHV1-18*01
IGHV1-18*04
IGHV1-2*02
IGHV1-24*01
IGHV1-3*02
IGHV1-46*01
IGHV1-58*01
IGHV1-58*02
IGHV1-69*01
IGHV1-69*04
IGHV1-69*06
IGHV1-69-2*01
IGHV2-26*01
IGHV2-5*01
IGHV2-5*02
IGHV2-70*01
IGHV2-70*04
IGHV2-70*15
IGHV3-11*05
IGHV3-11*06
IGHV3-13*04
IGHV3-13*05
IGHV3-15*01
IGHV3-20*04
IGHV3-21*01
IGHV3-23*01
IGHV3-23*04
IGHV3-30*18
IGHV3-30-3*01
IGHV3-33*01
IGHV3-43*01
IGHV3-48*01
IGHV3-48*02
IGHV3-49*03
IGHV3-49*05
IGHV3-53*01
IGHV3-53*02
IGHV3-64*01
IGHV3-64D*06
IGHV3-64D*06_S5429
IGHV3-66*01
IGHV3-7*03_S9833
IGHV3-7*04
IGHV3-72*01
IGHV3-73*01
IGHV3-73*02
IGHV3-74*01
IGHV4-28*01
IGHV4-30-2*01
IGHV4-30-4*01
IGHV4-31*03
IGHV4-34*01
IGHV4-39*01
IGHV4-4*02
IGHV4-4*07
IGHV4-59*01
IGHV4-59*08
IGHV4-61*01
IGHV5-10-1*01
IGHV5-10-1*03
IGHV5-51*01
IGHV5-51*03
IGHV6-1*01
V gene

ERR2567231

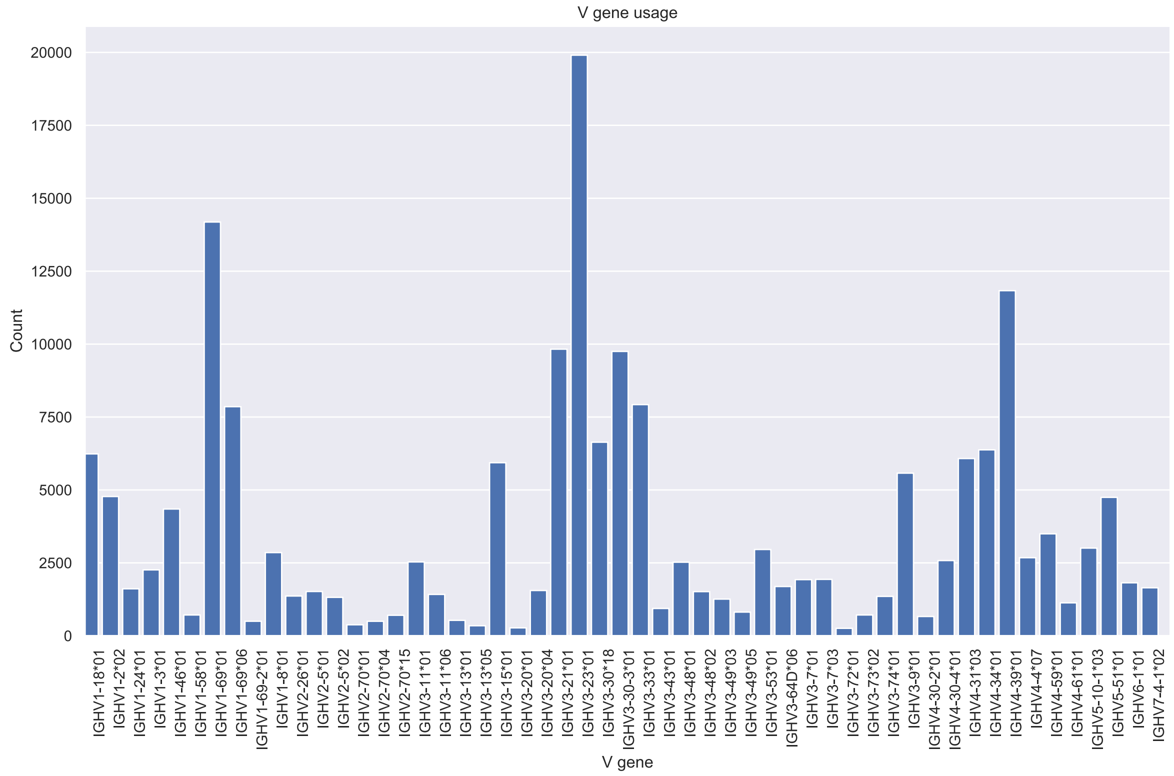
V gene usage
Count
20000
17500
15000
12500
10000
7500
5000
2500
0
IGHV1-18*01
IGHV1-2*02
IGHV1-24*01
IGHV1-3*01
IGHV1-46*01
IGHV1-58*01
IGHV1-69*01
IGHV1-69*06
IGHV1-69-2*01
IGHV1-8*01
IGHV2-26*01
IGHV2-5*01
IGHV2-5*02
IGHV2-70*01
IGHV2-70*04
IGHV2-70*15
IGHV3-11*01
IGHV3-11*06
IGHV3-13*01
IGHV3-13*05
IGHV3-15*01
IGHV3-20*01
IGHV3-20*04
IGHV3-21*01
IGHV3-23*01
IGHV3-30*18
IGHV3-30-3*01
IGHV3-33*01
IGHV3-43*01
IGHV3-48*01
IGHV3-48*02
IGHV3-49*03
IGHV3-49*05
IGHV3-53*01
IGHV3-64D*06
IGHV3-7*01
IGHV3-7*03
IGHV3-72*01
IGHV3-73*02
IGHV3-74*01
IGHV3-9*01
IGHV4-30-2*01
IGHV4-30-4*01
IGHV4-31*03
IGHV4-34*01
IGHV4-39*01
IGHV4-4*07
IGHV4-59*01
IGHV4-61*01
IGHV5-10-1*03
IGHV5-51*01
IGHV6-1*01
IGHV7-4-1*02
V gene

ERR2567232

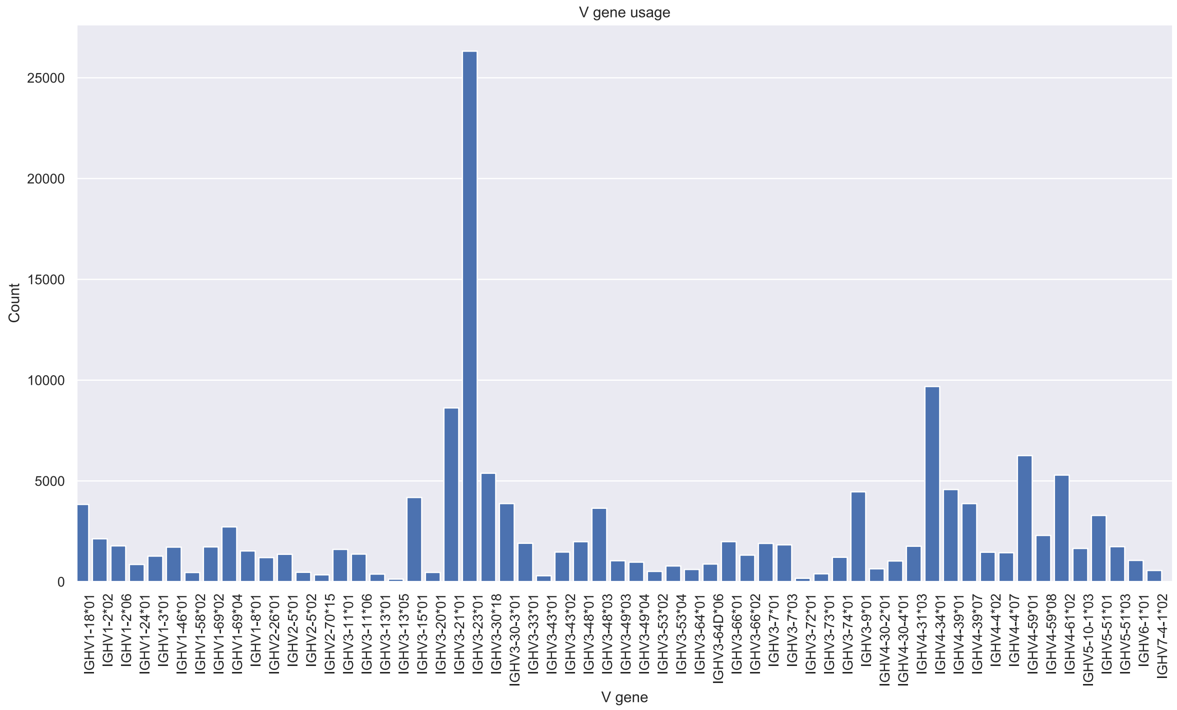

V gene usage
Count
25000
20000
15000
10000
5000
0
IGHV1-18*01
IGHV1-2*02
IGHV1-2*06
IGHV1-24*01
IGHV1-3*01
IGHV1-46*01
IGHV1-58*02
IGHV1-69*02
IGHV1-69*04
IGHV1-8*01
IGHV2-26*01
IGHV2-5*01
IGHV2-5*02
IGHV2-70*15
IGHV3-11*01
IGHV3-11*06
IGHV3-13*01
IGHV3-13*05
IGHV3-15*01
IGHV3-20*01
IGHV3-21*01
IGHV3-23*01
IGHV3-30*18
IGHV3-30-3*01
IGHV3-33*01
IGHV3-43*01
IGHV3-43*02
IGHV3-48*01
IGHV3-48*03
IGHV3-49*03
IGHV3-49*04
IGHV3-53*02
IGHV3-53*04
IGHV3-64*01
IGHV3-64D*06
IGHV3-66*01
IGHV3-66*02
IGHV3-7*01
IGHV3-7*03
IGHV3-72*01
IGHV3-73*01
IGHV3-74*01
IGHV3-9*01
IGHV4-30-2*01
IGHV4-30-4*01
IGHV4-31*03
IGHV4-34*01
IGHV4-39*01
IGHV4-39*07
IGHV4-4*02
IGHV4-4*07
IGHV4-59*01
IGHV4-59*08
IGHV4-61*02
IGHV5-10-1*03
IGHV5-51*01
IGHV5-51*03
IGHV6-1*01
IGHV7-4-1*02
V gene

ERR2567240

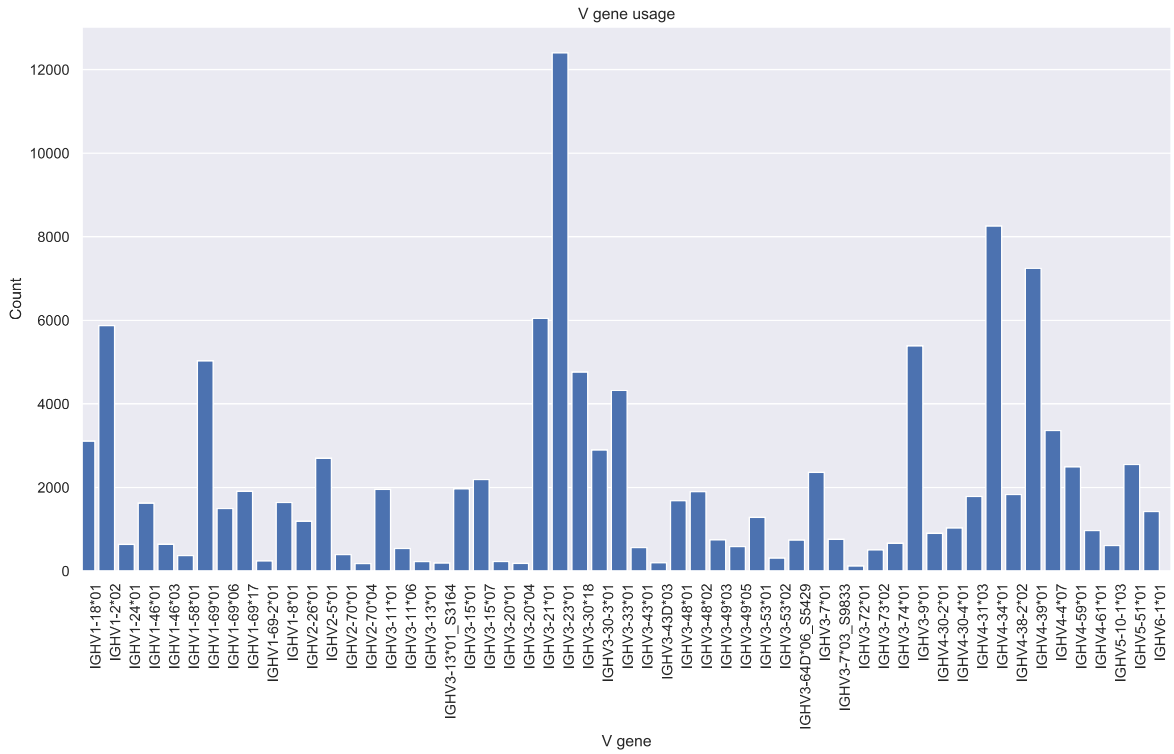

V gene usage
Count
0
2000
4000
6000
8000
10000
12000
V gene
IGHV1-18*01
IGHV1-2*02
IGHV1-24*01
IGHV1-46*01
IGHV1-46*03
IGHV1-58*01
IGHV1-69*01
IGHV1-69*06
IGHV1-69*17
IGHV1-69-2*01
IGHV1-8*01
IGHV2-26*01
IGHV2-5*01
IGHV2-70*01
IGHV2-70*04
IGHV3-11*01
IGHV3-11*06
IGHV3-13*01
IGHV3-13*01_S3164
IGHV3-15*01
IGHV3-15*07
IGHV3-20*01
IGHV3-20*04
IGHV3-21*01
IGHV3-23*01
IGHV3-30*18
IGHV3-30-3*01
IGHV3-33*01
IGHV3-43*01
IGHV3-43D*03
IGHV3-48*01
IGHV3-48*02
IGHV3-49*03
IGHV3-49*05
IGHV3-53*01
IGHV3-53*02
IGHV3-64D*06_S5429
IGHV3-7*01
IGHV3-7*03_S9833
IGHV3-72*01
IGHV3-73*02
IGHV3-74*01
IGHV3-9*01
IGHV4-30-2*01
IGHV4-30-4*01
IGHV4-31*03
IGHV4-34*01
IGHV4-38-2*02
IGHV4-39*01
IGHV4-4*07
IGHV4-59*01
IGHV4-61*01
IGHV5-10-1*03
IGHV5-51*01
IGHV6-1*01

ERR2567242

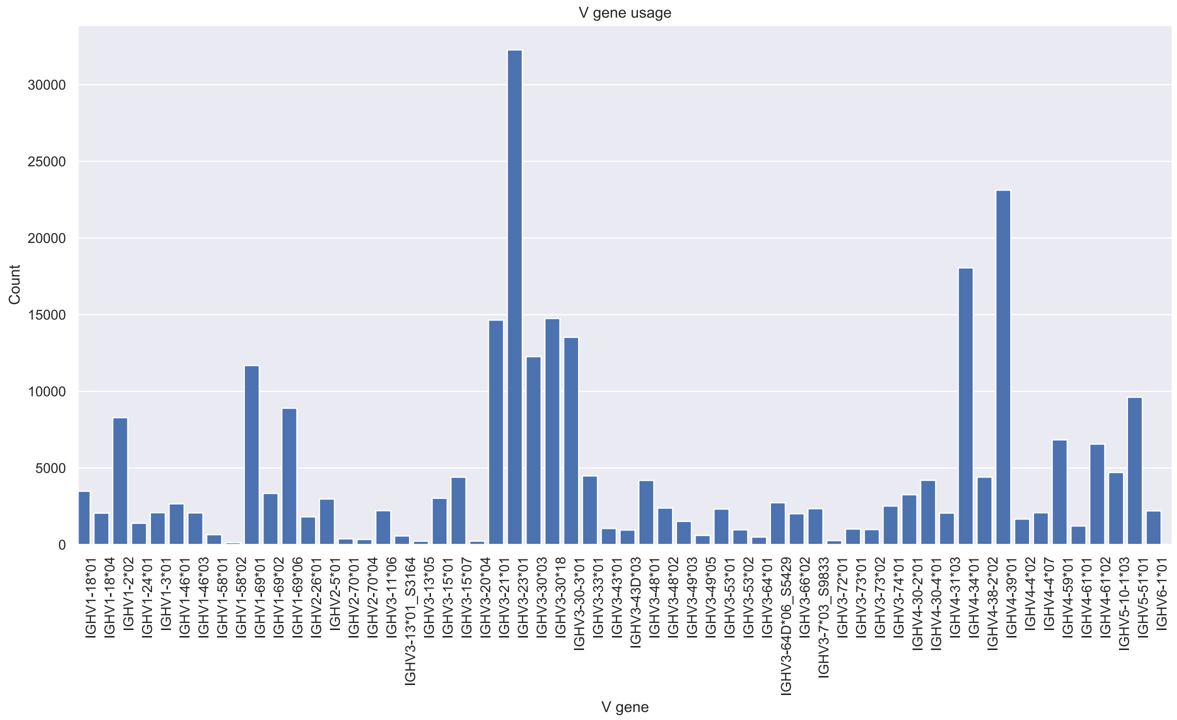

ERR2567243

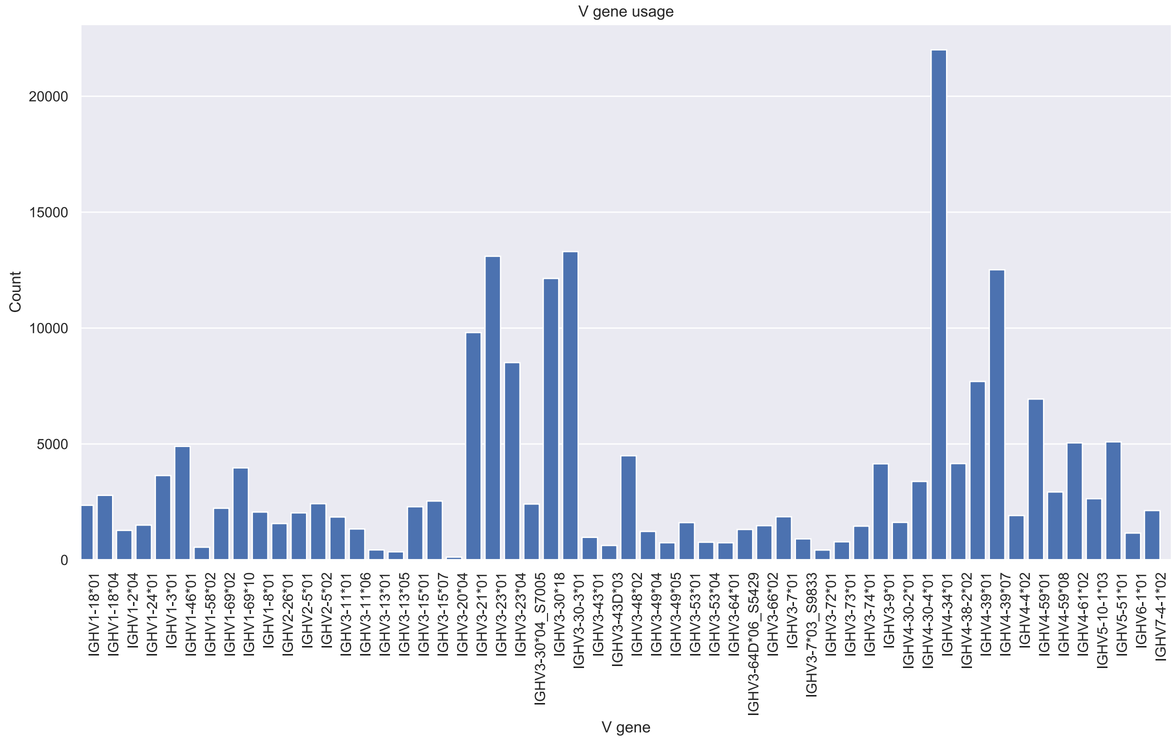
V gene usage
Count
V gene
0
5000
10000
15000
20000
IGHV1-18*01
IGHV1-18*04
IGHV1-2*04
IGHV1-24*01
IGHV1-3*01
IGHV1-46*01
IGHV1-58*02
IGHV1-69*02
IGHV1-69*10
IGHV1-8*01
IGHV2-26*01
IGHV2-5*01
IGHV2-5*02
IGHV3-11*01
IGHV3-11*06
IGHV3-13*01
IGHV3-13*05
IGHV3-15*01
IGHV3-15*07
IGHV3-20*04
IGHV3-21*01
IGHV3-23*01
IGHV3-23*04
IGHV3-30*04_S7005
IGHV3-30*18
IGHV3-30-3*01
IGHV3-43*01
IGHV3-43D*03
IGHV3-48*02
IGHV3-49*04
IGHV3-49*05
IGHV3-53*01
IGHV3-53*04
IGHV3-64*01
IGHV3-64D*06_S5429
IGHV3-66*02
IGHV3-7*01
IGHV3-7*03_S9833
IGHV3-72*01
IGHV3-73*01
IGHV3-74*01
IGHV3-9*01
IGHV4-30-2*01
IGHV4-30-4*01
IGHV4-34*01
IGHV4-38-2*02
IGHV4-39*01
IGHV4-39*07
IGHV4-4*02
IGHV4-59*01
IGHV4-59*08
IGHV4-61*02
IGHV5-10-1*03
IGHV5-51*01
IGHV6-1*01
IGHV7-4-1*02

ERR2567246

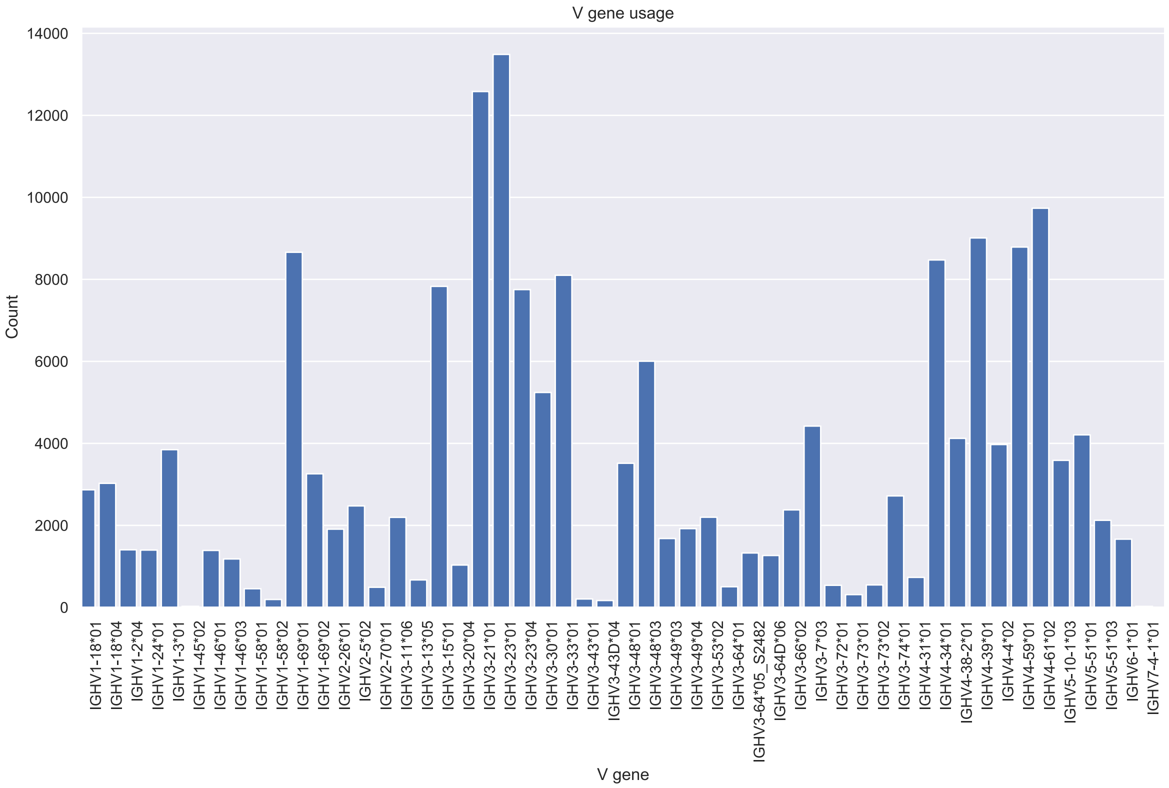
V gene usage
Count
V gene
0
2000
4000
6000
8000
10000
12000
14000
IGHV1-18*01
IGHV1-18*04
IGHV1-2*04
IGHV1-24*01
IGHV1-3*01
IGHV1-45*02
IGHV1-46*01
IGHV1-46*03
IGHV1-58*01
IGHV1-58*02
IGHV1-69*01
IGHV1-69*02
IGHV2-26*01
IGHV2-5*02
IGHV2-70*01
IGHV3-11*06
IGHV3-13*05
IGHV3-15*01
IGHV3-20*04
IGHV3-21*01
IGHV3-23*01
IGHV3-23*04
IGHV3-30*01
IGHV3-33*01
IGHV3-43*01
IGHV3-43D*04
IGHV3-48*01
IGHV3-48*03
IGHV3-49*03
IGHV3-49*04
IGHV3-53*02
IGHV3-64*01
IGHV3-64*05_S2482
IGHV3-64D*06
IGHV3-66*02
IGHV3-7*03
IGHV3-72*01
IGHV3-73*01
IGHV3-73*02
IGHV3-74*01
IGHV4-31*01
IGHV4-34*01
IGHV4-38-2*01
IGHV4-39*01
IGHV4-4*02
IGHV4-59*01
IGHV4-61*02
IGHV5-10-1*03
IGHV5-51*01
IGHV5-51*03
IGHV6-1*01
IGHV7-4-1*01

ERR2567249

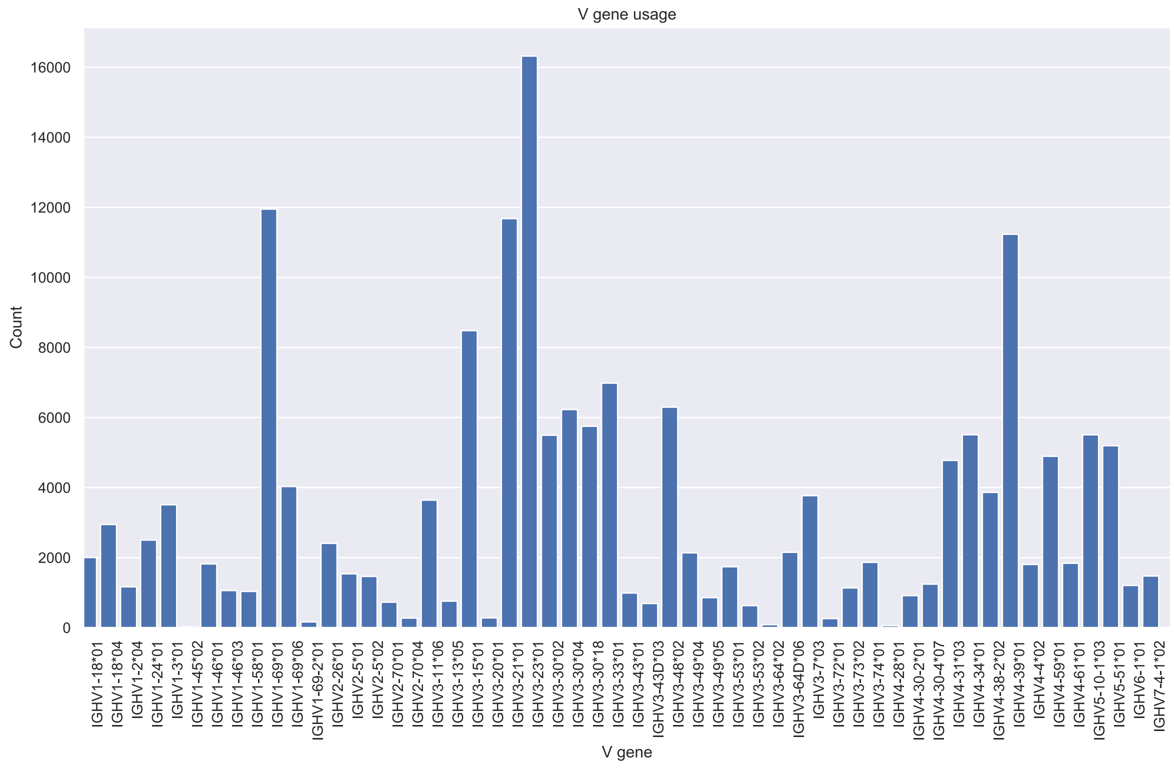
V gene usage
Count
V gene
0
2000
4000
6000
8000
10000
12000
14000
16000
IGHV1-18*01
IGHV1-18*04
IGHV1-2*04
IGHV1-24*01
IGHV1-3*01
IGHV1-45*02
IGHV1-46*01
IGHV1-46*03
IGHV1-58*01
IGHV1-69*01
IGHV1-69*06
IGHV1-69-2*01
IGHV2-26*01
IGHV2-5*01
IGHV2-5*02
IGHV2-70*01
IGHV2-70*04
IGHV3-11*06
IGHV3-13*05
IGHV3-15*01
IGHV3-20*01
IGHV3-21*01
IGHV3-23*01
IGHV3-30*02
IGHV3-30*04
IGHV3-30*18
IGHV3-33*01
IGHV3-43*01
IGHV3-43D*03
IGHV3-48*02
IGHV3-49*04
IGHV3-49*05
IGHV3-53*01
IGHV3-53*02
IGHV3-64*02
IGHV3-64D*06
IGHV3-7*03
IGHV3-72*01
IGHV3-73*02
IGHV3-74*01
IGHV4-28*01
IGHV4-30-2*01
IGHV4-30-4*07
IGHV4-31*03
IGHV4-34*01
IGHV4-38-2*02
IGHV4-39*01
IGHV4-4*02
IGHV4-59*01
IGHV4-61*01
IGHV5-10-1*03
IGHV5-51*01
IGHV6-1*01
IGHV7-4-1*02

ERR2567254

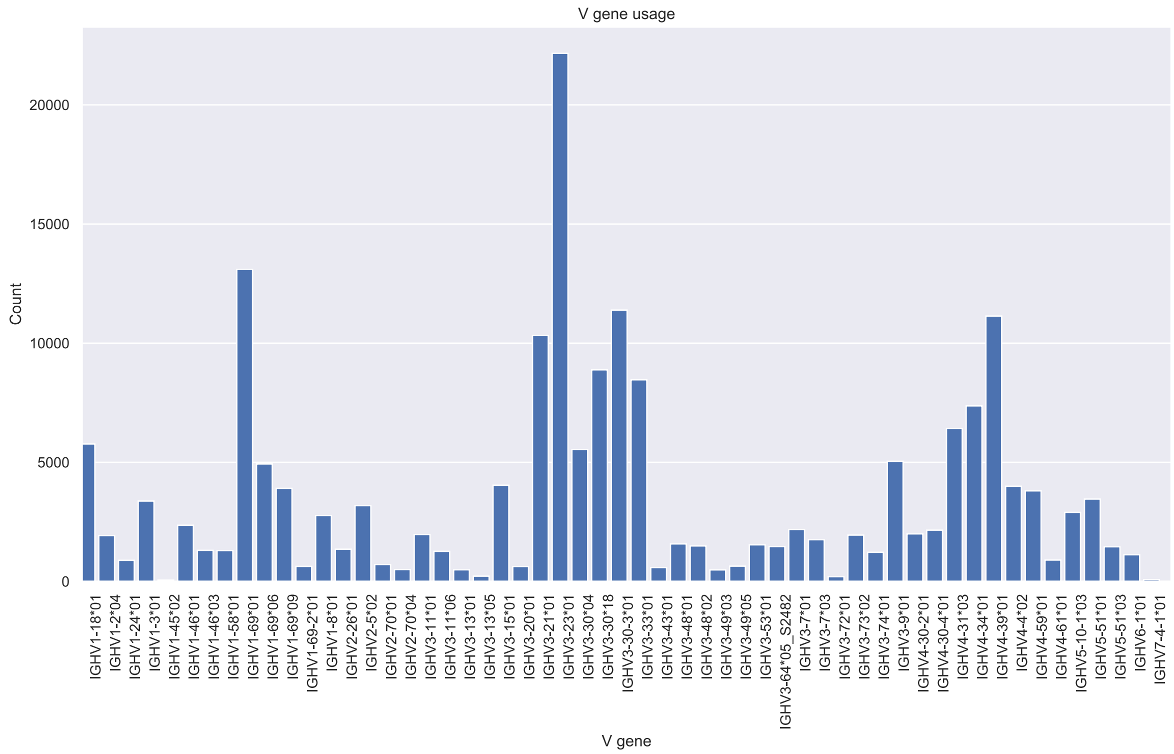
V gene usage
Count
0
5000
10000
15000
20000
V gene
IGHV1-18*01
IGHV1-2*04
IGHV1-24*01
IGHV1-3*01
IGHV1-45*02
IGHV1-46*01
IGHV1-46*03
IGHV1-58*01
IGHV1-69*01
IGHV1-69*06
IGHV1-69*09
IGHV1-69-2*01
IGHV1-8*01
IGHV2-26*01
IGHV2-5*02
IGHV2-70*01
IGHV2-70*04
IGHV3-11*01
IGHV3-11*06
IGHV3-13*01
IGHV3-13*05
IGHV3-15*01
IGHV3-20*01
IGHV3-21*01
IGHV3-23*01
IGHV3-30*04
IGHV3-30*18
IGHV3-30-3*01
IGHV3-33*01
IGHV3-43*01
IGHV3-48*01
IGHV3-48*02
IGHV3-49*03
IGHV3-49*05
IGHV3-53*01
IGHV3-64*05_S2482
IGHV3-7*01
IGHV3-7*03
IGHV3-72*01
IGHV3-73*02
IGHV3-74*01
IGHV3-9*01
IGHV4-30-2*01
IGHV4-30-4*01
IGHV4-31*03
IGHV4-34*01
IGHV4-39*01
IGHV4-4*02
IGHV4-59*01
IGHV4-61*01
IGHV5-10-1*03
IGHV5-51*01
IGHV5-51*03
IGHV6-1*01
IGHV7-4-1*01

ERR2567259

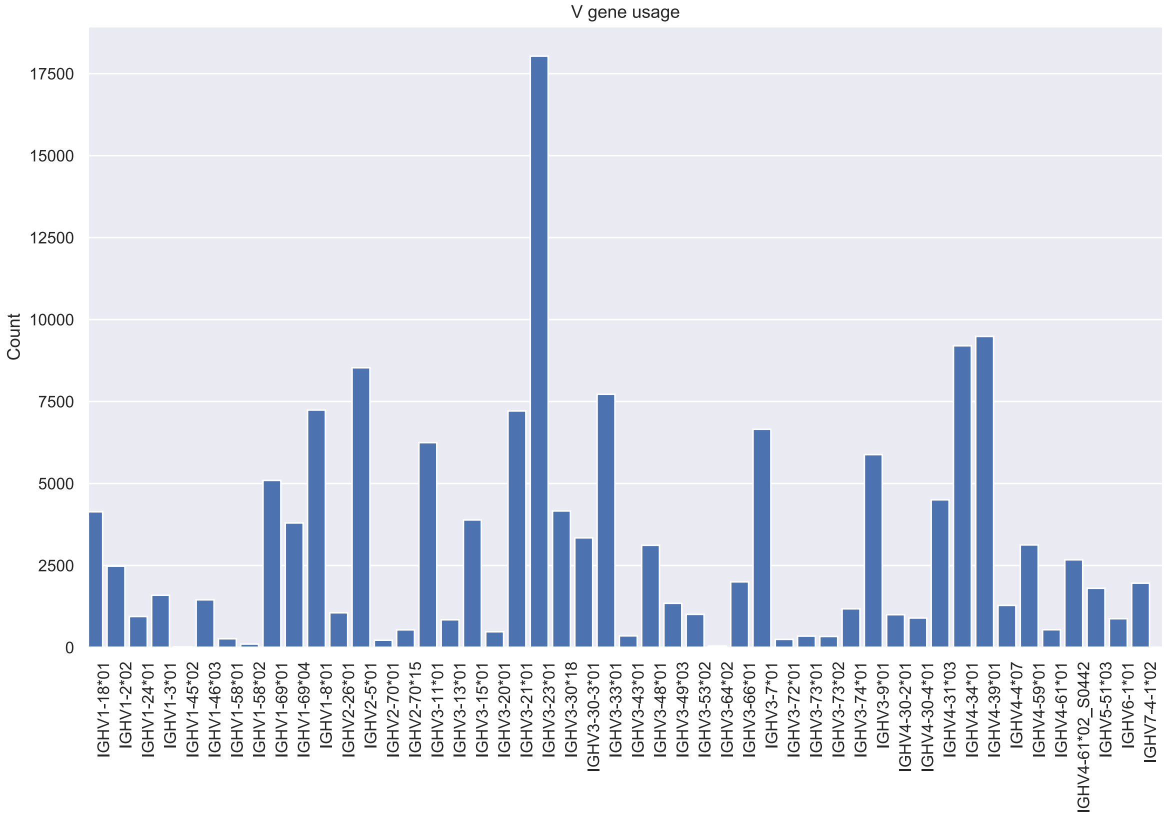
V gene usage
Count
0
2500
5000
7500
10000
12500
15000
17500
IGHV1-18*01
IGHV1-2*02
IGHV1-24*01
IGHV1-3*01
IGHV1-45*02
IGHV1-46*03
IGHV1-58*01
IGHV1-58*02
IGHV1-69*01
IGHV1-69*04
IGHV1-8*01
IGHV2-26*01
IGHV2-5*01
IGHV2-70*01
IGHV2-70*15
IGHV3-11*01
IGHV3-13*01
IGHV3-15*01
IGHV3-20*01
IGHV3-21*01
IGHV3-23*01
IGHV3-30*18
IGHV3-30-3*01
IGHV3-33*01
IGHV3-43*01
IGHV3-48*01
IGHV3-49*03
IGHV3-53*02
IGHV3-64*02
IGHV3-66*01
IGHV3-7*01
IGHV3-72*01
IGHV3-73*01
IGHV3-73*02
IGHV3-74*01
IGHV3-9*01
IGHV4-30-2*01
IGHV4-30-4*01
IGHV4-31*03
IGHV4-34*01
IGHV4-39*01
IGHV4-4*07
IGHV4-59*01
IGHV4-61*01
IGHV4-61*02_S0442
IGHV5-51*03
IGHV6-1*01
IGHV7-4-1*02
V gene

ERR2567261

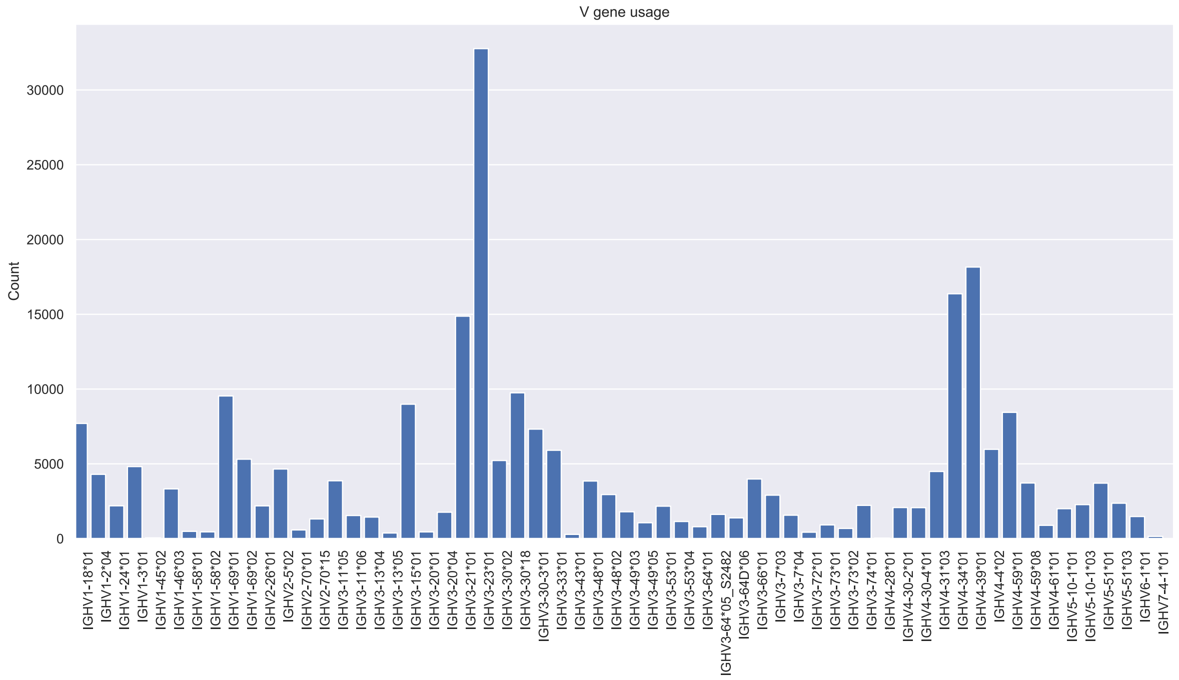

V gene usage
Count
30000
25000
20000
15000
10000
5000
0
IGHV1-18*01
IGHV1-2*04
IGHV1-24*01
IGHV1-3*01
IGHV1-45*02
IGHV1-46*03
IGHV1-58*01
IGHV1-58*02
IGHV1-69*01
IGHV1-69*02
IGHV2-26*01
IGHV2-5*02
IGHV2-70*01
IGHV2-70*15
IGHV3-11*05
IGHV3-11*06
IGHV3-13*04
IGHV3-13*05
IGHV3-15*01
IGHV3-20*01
IGHV3-20*04
IGHV3-21*01
IGHV3-23*01
IGHV3-30*02
IGHV3-30*18
IGHV3-30-3*01
IGHV3-33*01
IGHV3-43*01
IGHV3-48*01
IGHV3-48*02
IGHV3-49*03
IGHV3-49*05
IGHV3-53*01
IGHV3-53*04
IGHV3-64*01
IGHV3-64*05_S2482
IGHV3-64D*06
IGHV3-66*01
IGHV3-7*03
IGHV3-7*04
IGHV3-72*01
IGHV3-73*01
IGHV3-73*02
IGHV3-74*01
IGHV4-28*01
IGHV4-30-2*01
IGHV4-30-4*01
IGHV4-31*03
IGHV4-34*01
IGHV4-39*01
IGHV4-4*02
IGHV4-59*01
IGHV4-59*08
IGHV4-61*01
IGHV5-10-1*01
IGHV5-10-1*03
IGHV5-51*01
IGHV5-51*03
IGHV6-1*01
IGHV7-4-1*01
V gene

ERR2567263

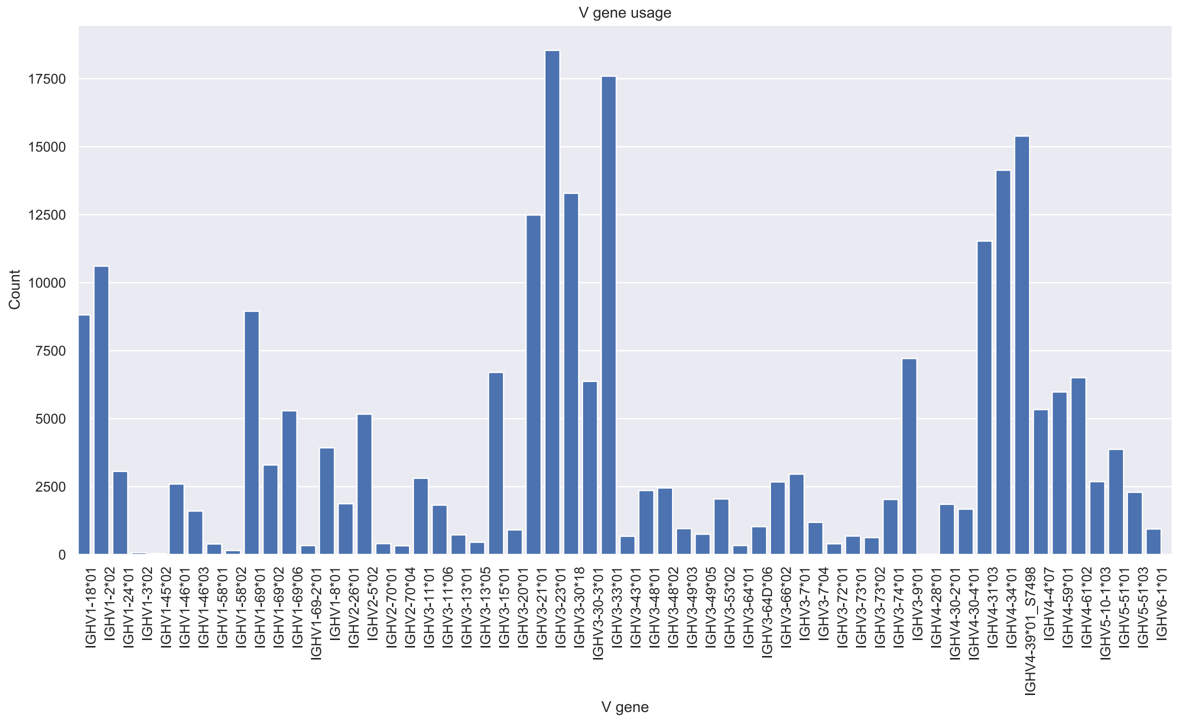
V gene usage
Count
17500
15000
12500
10000
7500
5000
2500
0
IGHV1-18*01
IGHV1-2*02
IGHV1-24*01
IGHV1-3*02
IGHV1-45*02
IGHV1-46*01
IGHV1-46*03
IGHV1-58*01
IGHV1-58*02
IGHV1-69*01
IGHV1-69*02
IGHV1-69*06
IGHV1-69-2*01
IGHV1-8*01
IGHV2-26*01
IGHV2-5*02
IGHV2-70*01
IGHV2-70*04
IGHV3-11*01
IGHV3-11*06
IGHV3-13*01
IGHV3-13*05
IGHV3-15*01
IGHV3-20*01
IGHV3-21*01
IGHV3-23*01
IGHV3-30*18
IGHV3-30-3*01
IGHV3-33*01
IGHV3-43*01
IGHV3-48*01
IGHV3-48*02
IGHV3-49*03
IGHV3-49*05
IGHV3-53*02
IGHV3-64*01
IGHV3-64D*06
IGHV3-66*02
IGHV3-7*01
IGHV3-7*04
IGHV3-72*01
IGHV3-73*01
IGHV3-73*02
IGHV3-74*01
IGHV3-9*01
IGHV4-28*01
IGHV4-30-2*01
IGHV4-30-4*01
IGHV4-31*03
IGHV4-34*01
IGHV4-39*01_S7498
IGHV4-4*07
IGHV4-59*01
IGHV4-61*02
IGHV5-10-1*03
IGHV5-51*01
IGHV5-51*03
IGHV6-1*01
V gene

ERR2567264

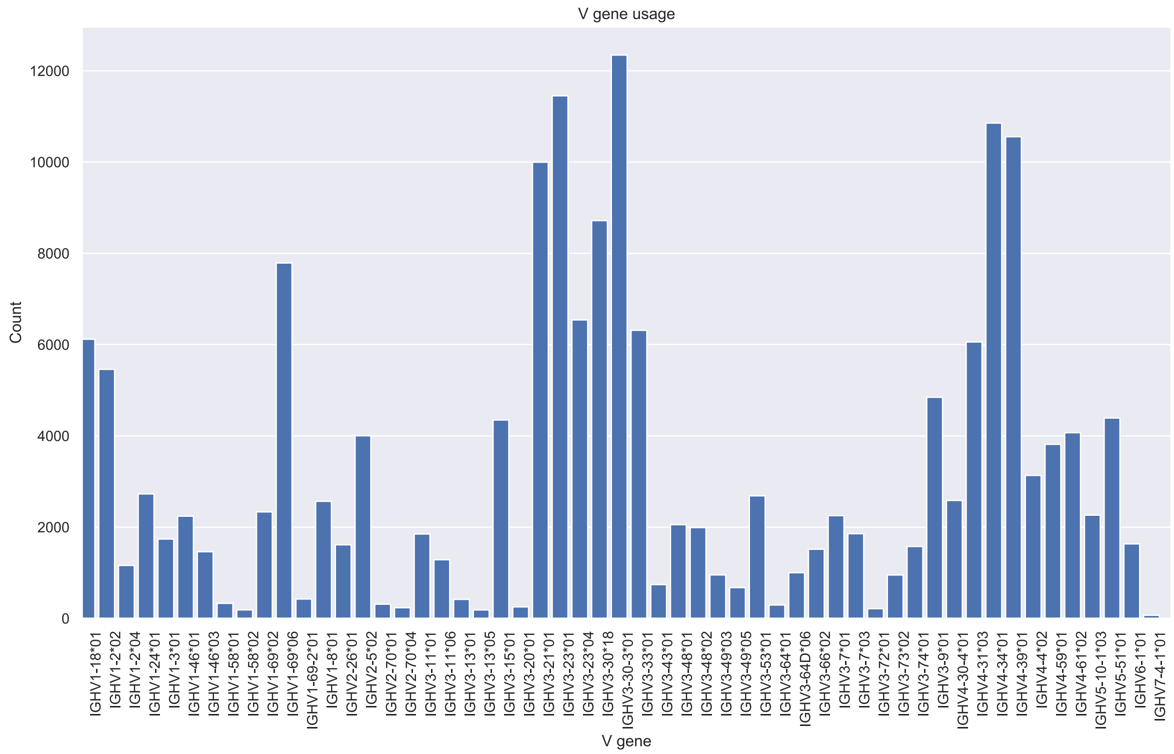
V gene usage
Count
0
2000
4000
6000
8000
10000
12000
V gene
IGHV1-18*01
IGHV1-2*02
IGHV1-2*04
IGHV1-24*01
IGHV1-3*01
IGHV1-46*01
IGHV1-46*03
IGHV1-58*01
IGHV1-58*02
IGHV1-69*02
IGHV1-69*06
IGHV1-69-2*01
IGHV1-8*01
IGHV2-26*01
IGHV2-5*02
IGHV2-70*01
IGHV2-70*04
IGHV3-11*01
IGHV3-11*06
IGHV3-13*01
IGHV3-13*05
IGHV3-15*01
IGHV3-20*01
IGHV3-21*01
IGHV3-23*01
IGHV3-23*04
IGHV3-30*18
IGHV3-30-3*01
IGHV3-33*01
IGHV3-43*01
IGHV3-48*01
IGHV3-48*02
IGHV3-49*03
IGHV3-49*05
IGHV3-53*01
IGHV3-64*01
IGHV3-64D*06
IGHV3-66*02
IGHV3-7*01
IGHV3-7*03
IGHV3-72*01
IGHV3-73*02
IGHV3-74*01
IGHV3-9*01
IGHV4-30-4*01
IGHV4-31*03
IGHV4-34*01
IGHV4-39*01
IGHV4-4*02
IGHV4-59*01
IGHV4-61*02
IGHV5-10-1*03
IGHV5-51*01
IGHV6-1*01
IGHV7-4-1*01

ERR2567265

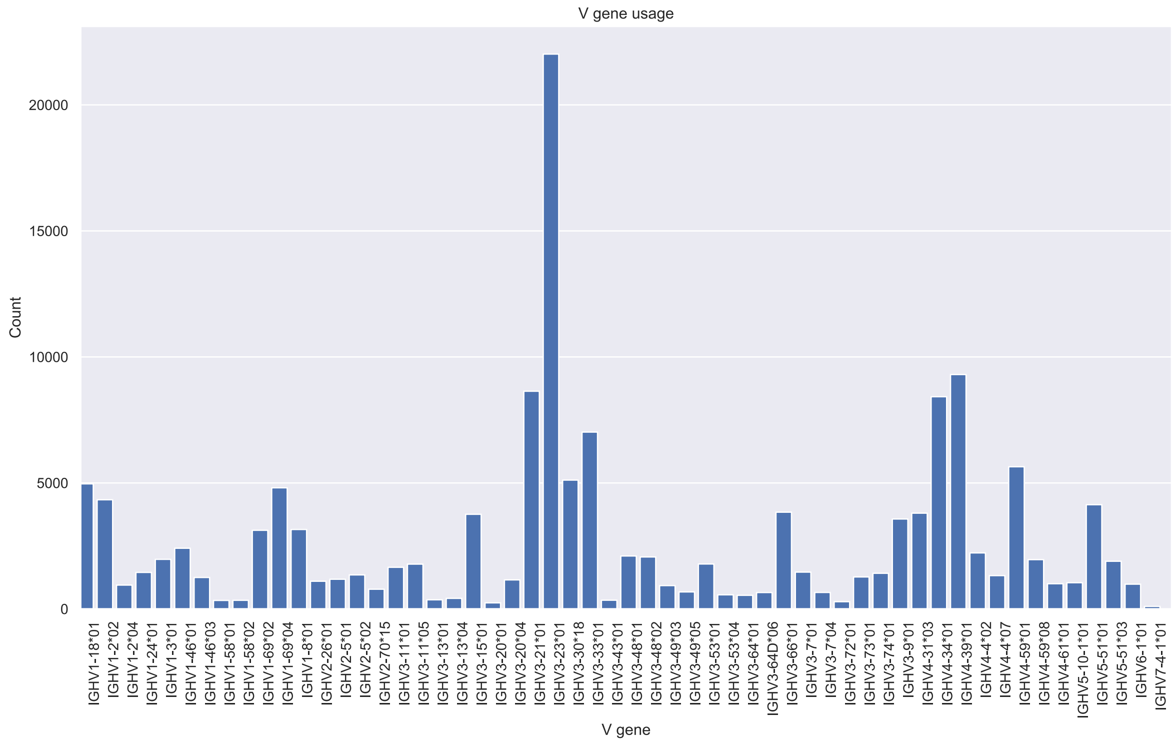
V gene usage
Count
0
5000
10000
15000
20000
V gene
IGHV1-18*01
IGHV1-2*02
IGHV1-2*04
IGHV1-24*01
IGHV1-3*01
IGHV1-46*01
IGHV1-46*03
IGHV1-58*01
IGHV1-58*02
IGHV1-69*02
IGHV1-69*04
IGHV1-8*01
IGHV2-26*01
IGHV2-5*01
IGHV2-5*02
IGHV2-70*15
IGHV3-11*01
IGHV3-11*05
IGHV3-13*01
IGHV3-13*04
IGHV3-15*01
IGHV3-20*01
IGHV3-20*04
IGHV3-21*01
IGHV3-23*01
IGHV3-30*18
IGHV3-33*01
IGHV3-43*01
IGHV3-48*01
IGHV3-48*02
IGHV3-49*03
IGHV3-49*05
IGHV3-53*01
IGHV3-53*04
IGHV3-64*01
IGHV3-64D*06
IGHV3-66*01
IGHV3-7*01
IGHV3-7*04
IGHV3-72*01
IGHV3-73*01
IGHV3-74*01
IGHV3-9*01
IGHV4-31*03
IGHV4-34*01
IGHV4-39*01
IGHV4-4*02
IGHV4-4*07
IGHV4-59*01
IGHV4-59*08
IGHV4-61*01
IGHV5-10-1*01
IGHV5-51*01
IGHV5-51*03
IGHV6-1*01
IGHV7-4-1*01

ERR2567266

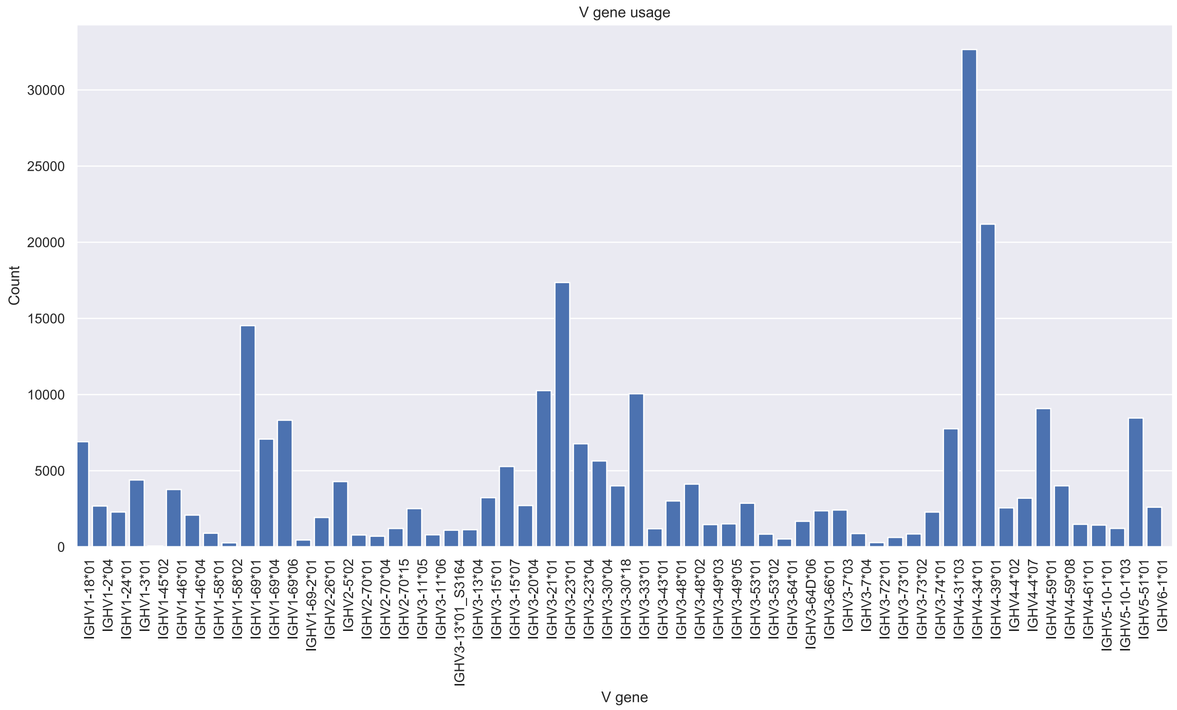
V gene usage
Count
0
5000
10000
15000
20000
25000
30000
V gene
IGHV1-18*01
IGHV1-2*04
IGHV1-24*01
IGHV1-3*01
IGHV1-45*02
IGHV1-46*01
IGHV1-46*04
IGHV1-58*01
IGHV1-58*02
IGHV1-69*01
IGHV1-69*04
IGHV1-69*06
IGHV1-69-2*01
IGHV2-26*01
IGHV2-5*02
IGHV2-70*01
IGHV2-70*04
IGHV2-70*15
IGHV3-11*05
IGHV3-11*06
IGHV3-13*01_S3164
IGHV3-13*04
IGHV3-15*01
IGHV3-15*07
IGHV3-20*04
IGHV3-21*01
IGHV3-23*01
IGHV3-23*04
IGHV3-30*04
IGHV3-30*18
IGHV3-33*01
IGHV3-43*01
IGHV3-48*01
IGHV3-48*02
IGHV3-49*03
IGHV3-49*05
IGHV3-53*01
IGHV3-53*02
IGHV3-64*01
IGHV3-64D*06
IGHV3-66*01
IGHV3-7*03
IGHV3-7*04
IGHV3-72*01
IGHV3-73*01
IGHV3-73*02
IGHV3-74*01
IGHV4-31*03
IGHV4-34*01
IGHV4-39*01
IGHV4-4*02
IGHV4-4*07
IGHV4-59*01
IGHV4-59*08
IGHV4-61*01
IGHV5-10-1*01
IGHV5-10-1*03
IGHV5-51*01
IGHV6-1*01

ERR2567271

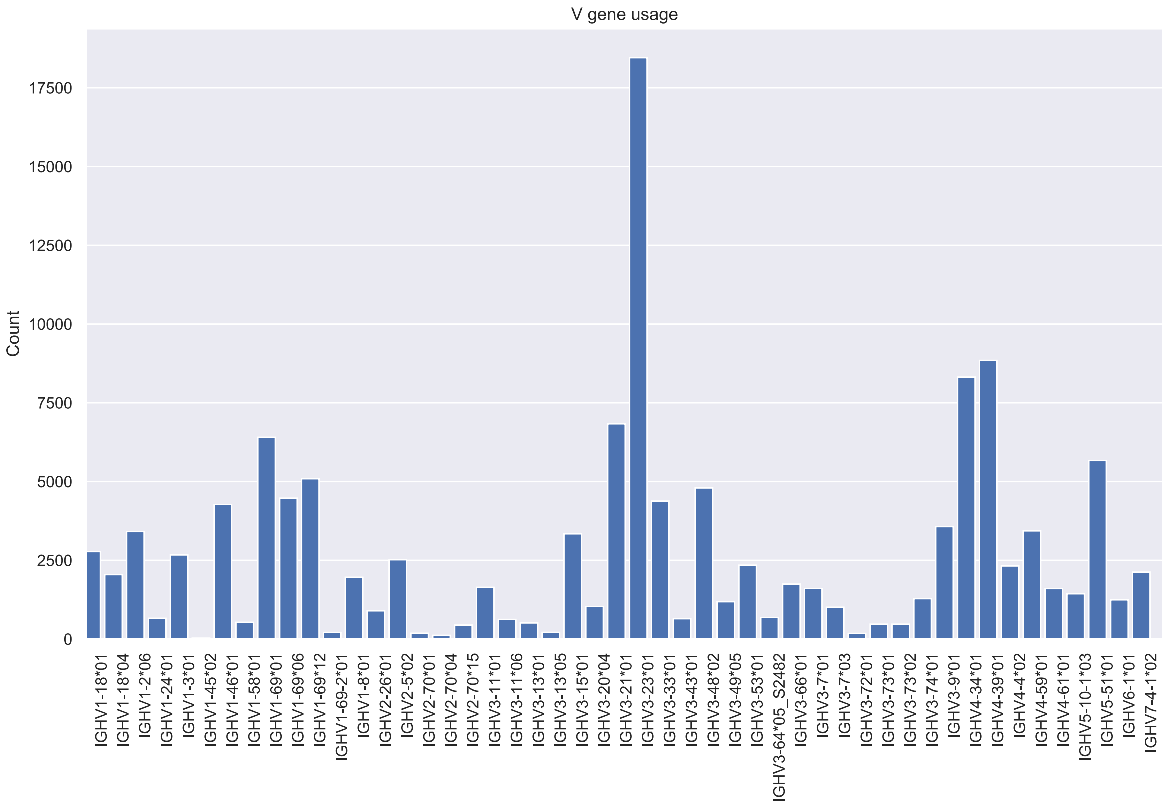
V gene usage
Count
17500
15000
12500
10000
7500
5000
2500
0
IGHV1-18*01
IGHV1-18*04
IGHV1-2*06
IGHV1-24*01
IGHV1-3*01
IGHV1-45*02
IGHV1-46*01
IGHV1-58*01
IGHV1-69*01
IGHV1-69*06
IGHV1-69*12
IGHV1-69-2*01
IGHV1-8*01
IGHV2-26*01
IGHV2-5*02
IGHV2-70*01
IGHV2-70*04
IGHV2-70*15
IGHV3-11*01
IGHV3-11*06
IGHV3-13*01
IGHV3-13*05
IGHV3-15*01
IGHV3-20*04
IGHV3-21*01
IGHV3-23*01
IGHV3-33*01
IGHV3-43*01
IGHV3-48*02
IGHV3-49*05
IGHV3-53*01
IGHV3-64*05_S2482
IGHV3-66*01
IGHV3-7*01
IGHV3-7*03
IGHV3-72*01
IGHV3-73*01
IGHV3-73*02
IGHV3-74*01
IGHV3-9*01
IGHV4-34*01
IGHV4-39*01
IGHV4-4*02
IGHV4-59*01
IGHV4-61*01
IGHV5-10-1*03
IGHV5-51*01
IGHV6-1*01
IGHV7-4-1*02
V gene

ERR2567274

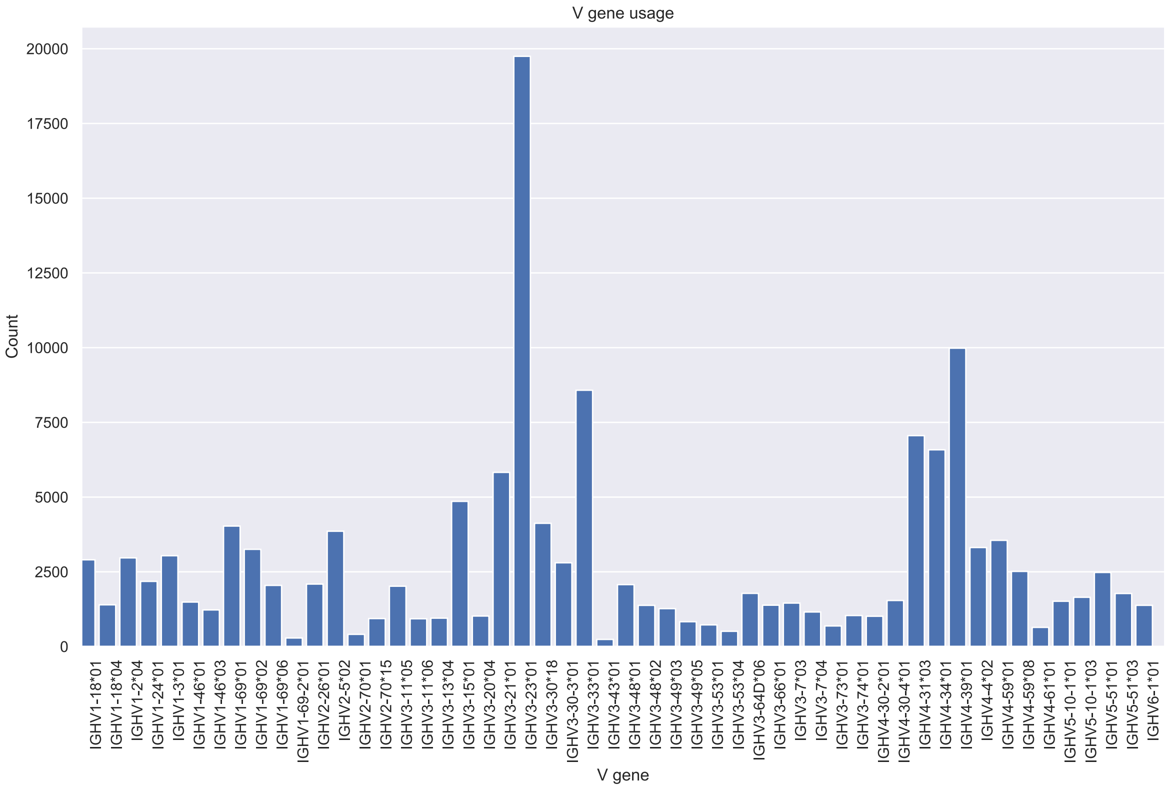
V gene usage
Count
0
2500
5000
7500
10000
12500
15000
17500
20000
V gene
IGHV1-18*01
IGHV1-18*04
IGHV1-2*04
IGHV1-24*01
IGHV1-3*01
IGHV1-46*01
IGHV1-46*03
IGHV1-69*01
IGHV1-69*02
IGHV1-69*06
IGHV1-69-2*01
IGHV2-26*01
IGHV2-5*02
IGHV2-70*01
IGHV2-70*15
IGHV3-11*05
IGHV3-11*06
IGHV3-13*04
IGHV3-15*01
IGHV3-20*04
IGHV3-21*01
IGHV3-23*01
IGHV3-30*18
IGHV3-30-3*01
IGHV3-33*01
IGHV3-43*01
IGHV3-48*01
IGHV3-48*02
IGHV3-49*03
IGHV3-49*05
IGHV3-53*01
IGHV3-53*04
IGHV3-64D*06
IGHV3-66*01
IGHV3-7*03
IGHV3-7*04
IGHV3-73*01
IGHV3-74*01
IGHV4-30-2*01
IGHV4-30-4*01
IGHV4-31*03
IGHV4-34*01
IGHV4-39*01
IGHV4-4*02
IGHV4-59*01
IGHV4-59*08
IGHV4-61*01
IGHV5-10-1*01
IGHV5-10-1*03
IGHV5-51*01
IGHV5-51*03
IGHV6-1*01

ERR2567276

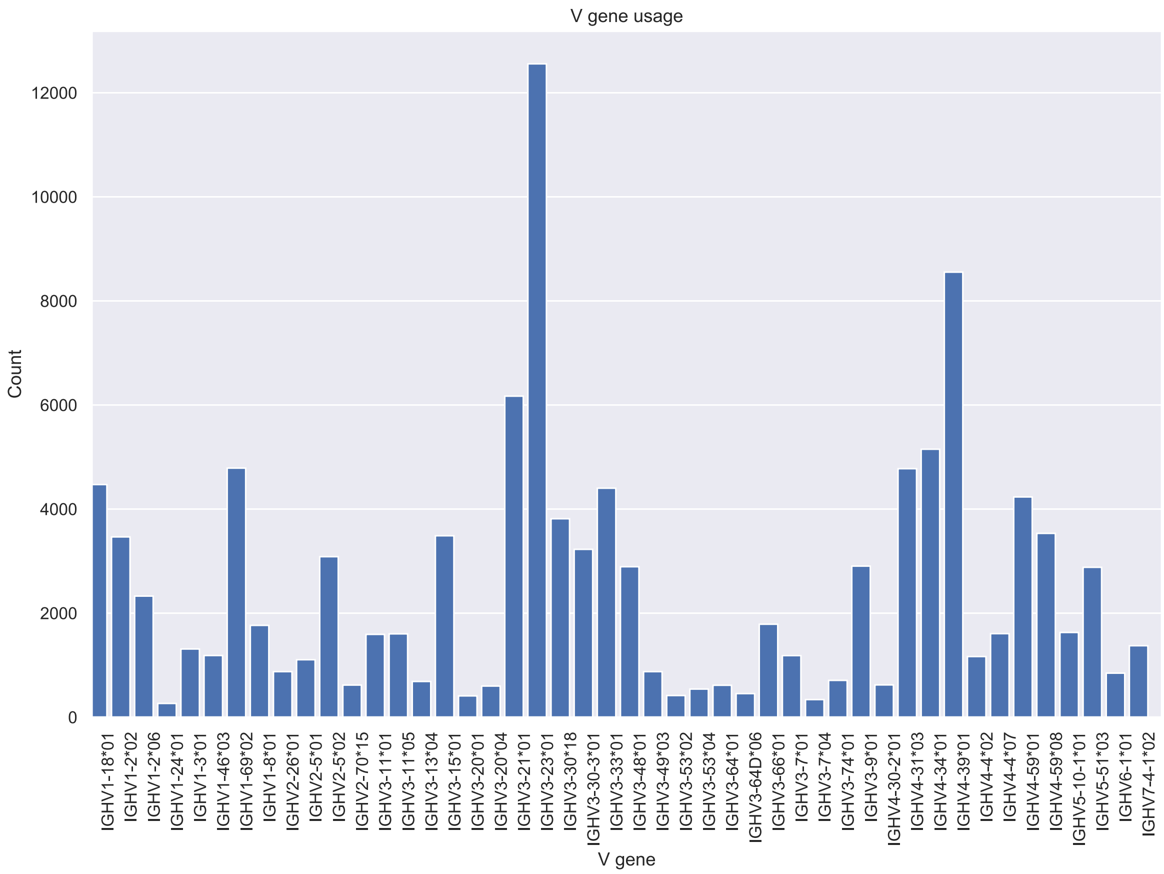

V gene usage
Count
0
2000
4000
6000
8000
10000
12000
V gene
IGHV1-18*01
IGHV1-2*02
IGHV1-2*06
IGHV1-24*01
IGHV1-3*01
IGHV1-46*03
IGHV1-69*02
IGHV1-8*01
IGHV2-26*01
IGHV2-5*01
IGHV2-5*02
IGHV2-70*15
IGHV3-11*01
IGHV3-11*05
IGHV3-13*04
IGHV3-15*01
IGHV3-20*01
IGHV3-20*04
IGHV3-21*01
IGHV3-23*01
IGHV3-30*18
IGHV3-30-3*01
IGHV3-33*01
IGHV3-48*01
IGHV3-49*03
IGHV3-53*02
IGHV3-53*04
IGHV3-64*01
IGHV3-64D*06
IGHV3-66*01
IGHV3-7*01
IGHV3-7*04
IGHV3-74*01
IGHV3-9*01
IGHV4-30-2*01
IGHV4-31*03
IGHV4-34*01
IGHV4-39*01
IGHV4-4*02
IGHV4-4*07
IGHV4-59*01
IGHV4-59*08
IGHV5-10-1*01
IGHV5-51*03
IGHV6-1*01
IGHV7-4-1*02

ERR2567277

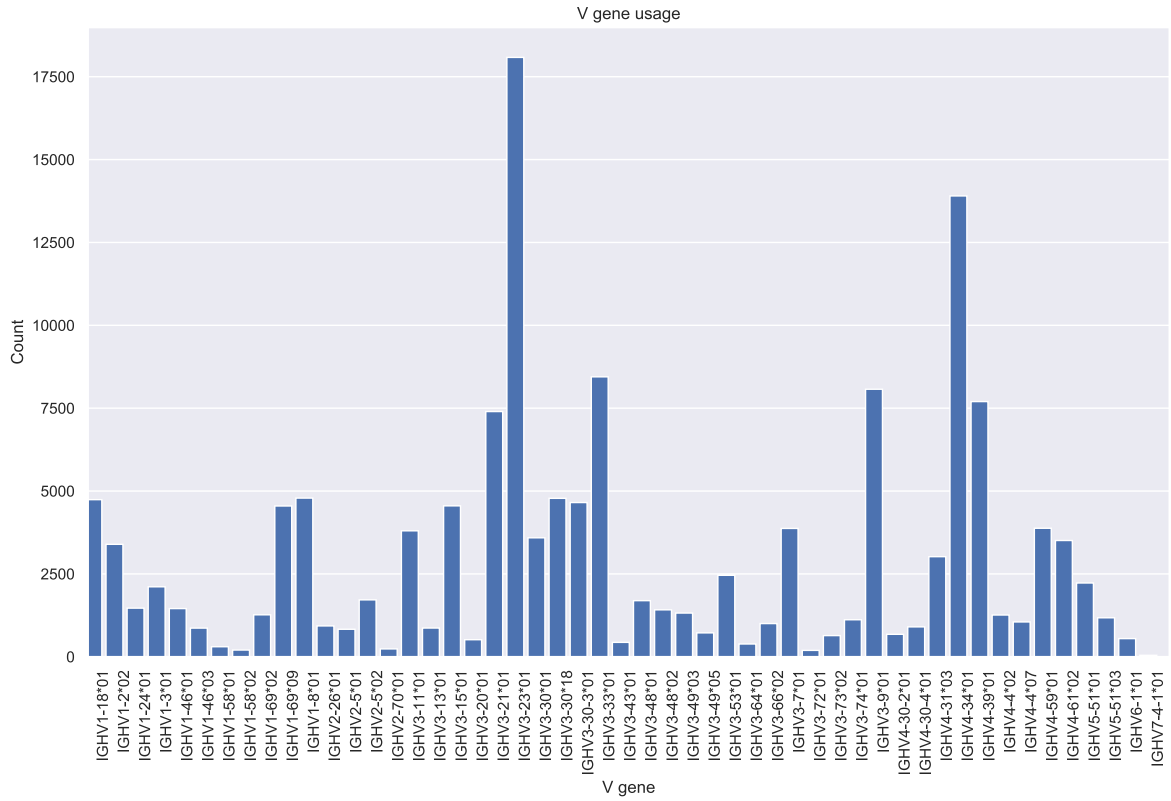
V gene usage
Count
17500
15000
12500
10000
7500
5000
2500
0
V gene
IGHV1-18*01
IGHV1-2*02
IGHV1-24*01
IGHV1-3*01
IGHV1-46*01
IGHV1-46*03
IGHV1-58*01
IGHV1-58*02
IGHV1-69*02
IGHV1-69*09
IGHV1-8*01
IGHV2-26*01
IGHV2-5*01
IGHV2-5*02
IGHV2-70*01
IGHV3-11*01
IGHV3-13*01
IGHV3-15*01
IGHV3-20*01
IGHV3-21*01
IGHV3-23*01
IGHV3-30*01
IGHV3-30*18
IGHV3-30-3*01
IGHV3-33*01
IGHV3-43*01
IGHV3-48*01
IGHV3-48*02
IGHV3-49*03
IGHV3-49*05
IGHV3-53*01
IGHV3-64*01
IGHV3-66*02
IGHV3-7*01
IGHV3-72*01
IGHV3-73*02
IGHV3-74*01
IGHV3-9*01
IGHV4-30-2*01
IGHV4-30-4*01
IGHV4-31*03
IGHV4-34*01
IGHV4-39*01
IGHV4-4*02
IGHV4-4*07
IGHV4-59*01
IGHV4-61*02
IGHV5-51*01
IGHV5-51*03
IGHV6-1*01
IGHV7-4-1*01
