# Supplementary material for: Poorly Expressed Alleles of Several Human Immunoglobulin Heavy Chain Variable Genes are Common in the Human Population
Source: Front Immunol. 2021 Feb 24;11:603980. doi: 10.3389/fimmu.2020.603980 (PMC7943739; doi:10.3389/fimmu.2020.603980)
Supplement: Supplementary Figure 3 — Allelic variants of IGHV1-2 as defined by IMGT are illustrated. Variability of some of the positions of these genes in samples obtained in different geographical locations as illustrated by the ENSEMBL browser (release 101, August 2020) (21) is shown. Only bases 163, 223, and 299 [IMGT numbering nomenclature (20)] of this gene display frequencies of variation >1% in the 1000 Genomes Project. The variant (SNP rs12588974) at base 299, indicative of the IGHV1-2*01 or IGHV1-2*05 alleles is present at about 5% in European populations. Bases 233 and 234 (SNPs rs782139757 and rs1425538657), that separate these two alleles remains as T and G, respectively, at very high frequency in most populations suggesting that IGHV1-2*01 is not common in these populations (not shown). All sequence variants of the illustrations of SNPs are indicated as seen in the reversed strand, hence they are complementary to the base of the coding strand. [file Image_3.pdf]

Supplementary Figure 3. Allelic variants of IGHV1-2 as defined by IMGT are illustrated. Variability of some of the positions of these genes in samples obtained in different geographical locations as illustrated by the ENSEMBL browser (release 101, August 2020) (Yates et al., 2020) is shown. Only bases 163, 223, and 299 (IMGT numbering nomenclature (Lefranc, 2011)) of this gene display frequencies of variation >1% in the 1000 Genomes Project. The variant (SNP rs12588974) at base 299, indicative of the IGHV1-2*01 or IGHV1-2*05 alleles is present at about 5% in European populations. Bases 233 and 234 (SNPs rs782139757 and rs1425538657), that separate these two alleles remains as T and G, respectively, at very high frequency in most populations suggesting that IGHV1-2*01 is not common in these populations (not shown). All sequence variants of the illustrations of SNPs are indicated as seen in the reversed strand, hence they are complementary to the base of the coding strand.

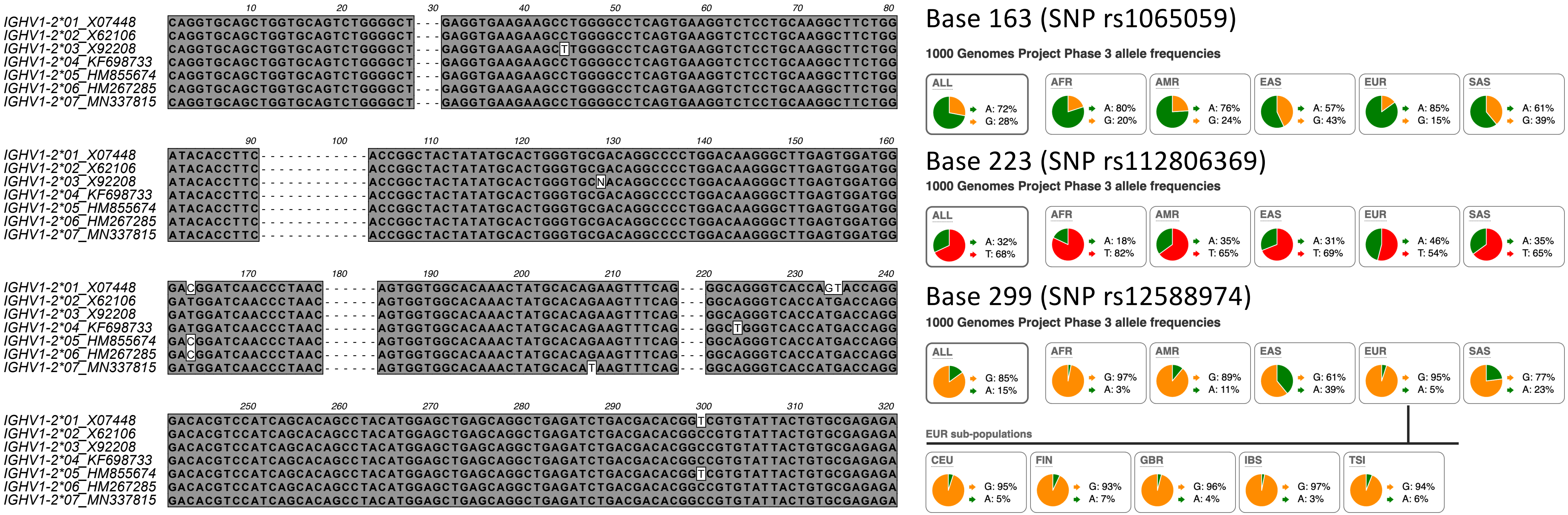

10 20 30 40 50 60 70 80
IGHV1-2*01_X07448 CAGGTGCAGCTGGTGCAGTCTGGGGCT---GAGGTGAAGAAGCCTGGGGCCTCAGTGAAGGTCTCCTGCAAGGCTTCTGG
IGHV1-2*02_X62106 CAGGTGCAGCTGGTGCAGTCTGGGGCT---GAGGTGAAGAAGCCTGGGGCCTCAGTGAAGGTCTCCTGCAAGGCTTCTGG
IGHV1-2*03_X92208 CAGGTGCAGCTGGTGCAGTCTGGGGCT---GAGGTGAAGAAGCTTGGGGCCTCAGTGAAGGTCTCCTGCAAGGCTTCTGG
IGHV1-2*04_KF698733 CAGGTGCAGCTGGTGCAGTCTGGGGCT---GAGGTGAAGAAGCCTGGGGCCTCAGTGAAGGTCTCCTGCAAGGCTTCTGG
IGHV1-2*05_HM855674 CAGGTGCAGCTGGTGCAGTCTGGGGCT---GAGGTGAAGAAGCCTGGGGCCTCAGTGAAGGTCTCCTGCAAGGCTTCTGG
IGHV1-2*06_HM267285 CAGGTGCAGCTGGTGCAGTCTGGGGCT---GAGGTGAAGAAGCCTGGGGCCTCAGTGAAGGTCTCCTGCAAGGCTTCTGG
IGHV1-2*07_MN337815 CAGGTGCAGCTGGTGCAGTCTGGGGCT---GAGGTGAAGAAGCCTGGGGCCTCAGTGAAGGTCTCCTGCAAGGCTTCTGG
90 100 110 120 130 140 150 160
IGHV1-2*01_X07448 ATACACCTTC------------ACCGGCTACTATATGCACTGGGTGCGACAGGCCCCTGGACAAGGGCTTGAGTGGATGG
IGHV1-2*02_X62106 ATACACCTTC------------ACCGGCTACTATATGCACTGGGTGCGACAGGCCCCTGGACAAGGGCTTGAGTGGATGG
IGHV1-2*03_X92208 ATACACCTTC------------ACCGGCTACTATATGCACTGGGTGCNACAGGCCCCTGGACAAGGGCTTGAGTGGATGG
IGHV1-2*04_KF698733 ATACACCTTC------------ACCGGCTACTATATGCACTGGGTGCGACAGGCCCCTGGACAAGGGCTTGAGTGGATGG
IGHV1-2*05_HM855674 ATACACCTTC------------ACCGGCTACTATATGCACTGGGTGCGACAGGCCCCTGGACAAGGGCTTGAGTGGATGG
IGHV1-2*06_HM267285 ATACACCTTC------------ACCGGCTACTATATGCACTGGGTGCGACAGGCCCCTGGACAAGGGCTTGAGTGGATGG
IGHV1-2*07_MN337815 ATACACCTTC------------ACCGGCTACTATATGCACTGGGTGCGACAGGCCCCTGGACAAGGGCTTGAGTGGATGG
170 180 190 200 210 220 230 240
IGHV1-2*01_X07448 GACGGATCAACCCTAAC------AGTGGTGGCACAAACTATGCACAGAAGTTTCAG---GGCAGGGTCACCAGTACCAGG
IGHV1-2*02_X62106 GATGGATCAACCCTAAC------AGTGGTGGCACAAACTATGCACAGAAGTTTCAG---GGCAGGGTCACCATGACCAGG
IGHV1-2*03_X92208 GATGGATCAACCCTAAC------AGTGGTGGCACAAACTATGCACAGAAGTTTCAG---GGCAGGGTCACCATGACCAGG
IGHV1-2*04_KF698733 GATGGATCAACCCTAAC------AGTGGTGGCACAAACTATGCACAGAAGTTTCAG---GGCTGGGTCACCATGACCAGG
IGHV1-2*05_HM855674 GACGGATCAACCCTAAC------AGTGGTGGCACAAACTATGCACAGAAGTTTCAG---GGCAGGGTCACCATGACCAGG
IGHV1-2*06_HM267285 GACGGATCAACCCTAAC------AGTGGTGGCACAAACTATGCACAGAAGTTTCAG---GGCAGGGTCACCATGACCAGG
IGHV1-2*07_MN337815 GATGGATCAACCCTAAC------AGTGGTGGCACAAACTATGCACATAAGTTTCAG---GGCAGGGTCACCATGACCAGG
250 260 270 280 290 300 310 320
IGHV1-2*01_X07448 GACACGTCCATCAGCACAGCCTACATGGAGCTGAGCAGGCTGAGATCTGACGACACGGTCGTGTATTACTGTGCGAGAGA
IGHV1-2*02_X62106 GACACGTCCATCAGCACAGCCTACATGGAGCTGAGCAGGCTGAGATCTGACGACACGGCCGTGTATTACTGTGCGAGAGA
IGHV1-2*03_X92208 GACACGTCCATCAGCACAGCCTACATGGAGCTGAGCAGGCTGAGATCTGACGACACGGCCGTGTATTACTGTGCGAGAGA
IGHV1-2*04_KF698733 GACACGTCCATCAGCACAGCCTACATGGAGCTGAGCAGGCTGAGATCTGACGACACGGCCGTGTATTACTGTGCGAGAGA
IGHV1-2*05_HM855674 GACACGTCCATCAGCACAGCCTACATGGAGCTGAGCAGGCTGAGATCTGACGACACGGTCGTGTATTACTGTGCGAGAGA
IGHV1-2*06_HM267285 GACACGTCCATCAGCACAGCCTACATGGAGCTGAGCAGGCTGAGATCTGACGACACGGCCGTGTATTACTGTGCGAGAGA
IGHV1-2*07_MN337815 GACACGTCCATCAGCACAGCCTACATGGAGCTGAGCAGGCTGAGATCTGACGACACGGCCGTGTATTACTGTGCGAGAGA
Base 163 (SNP rs1065059)
1000 Genomes Project Phase 3 allele frequencies
ALL A: 72% G: 28%
AFR A: 80% G: 20%
AMR A: 76% G: 24%
EAS A: 57% G: 43%
EUR A: 85% G: 15%
SAS A: 61% G: 39%
Base 223 (SNP rs112806369)
1000 Genomes Project Phase 3 allele frequencies
ALL A: 32% T: 68%
AFR A: 18% T: 82%
AMR A: 35% T: 65%
EAS A: 31% T: 69%
EUR A: 46% T: 54%
SAS A: 35% T: 65%
Base 299 (SNP rs12588974)
1000 Genomes Project Phase 3 allele frequencies
ALL G: 85% A: 15%
AFR G: 97% A: 3%
AMR G: 89% A: 11%
EAS G: 61% A: 39%
EUR G: 95% A: 5%
SAS G: 77% A: 23%
EUR sub-populations
CEU G: 95% A: 5%
FIN G: 93% A: 7%
GBR G: 96% A: 4%
IBS G: 97% A: 3%
TSI G: 94% A: 6%
