# Supplementary material for: Poorly Expressed Alleles of Several Human Immunoglobulin Heavy Chain Variable Genes are Common in the Human Population
Source: Front Immunol. 2021 Feb 24;11:603980. doi: 10.3389/fimmu.2020.603980 (PMC7943739; doi:10.3389/fimmu.2020.603980)
Supplement: Supplementary Figure 4 — Allelic variants of IGHV1-3 as defined by IMGT are illustrated. Variability of some of the positions of these genes in samples obtained in different geographical locations as illustrated by the ENSEMBL browser (release 101, August 2020) (21) is shown. Only bases 6, 12, 167, 208, 291 and 296 [IMGT numbering nomenclature (20)] of this gene display frequencies of variation >1% in the 1000 Genomes Project. Variants indicative of the IGHV1-3*02 allele are present at about 40%. All sequence variants of the illustrations of SNPs are indicated as seen in the reversed strand, hence they are complementary to the base of the coding strand. [file Image_4.pdf]

Supplementary Figure 4. Allelic variants of IGHV1-3 as defined by IMGT are illustrated. Variability of some of the positions of these genes in samples obtained in different geographical locations as illustrated by the ENSEMBL browser (release 101, August 2020) (Yates et al., 2020) is shown. Only bases 6, 12, 167, 208, 291 and 296 (IMGT numbering nomenclature (Lefranc, 2011)) of this gene display frequencies of variation >1% in the 1000 Genomes Project. Variants indicative of the IGHV1-3*02 allele are present at about 40%. All sequence variants of the illustrations of SNPs are indicated as seen in the reversed strand, hence they are complementary to the base of the coding strand.

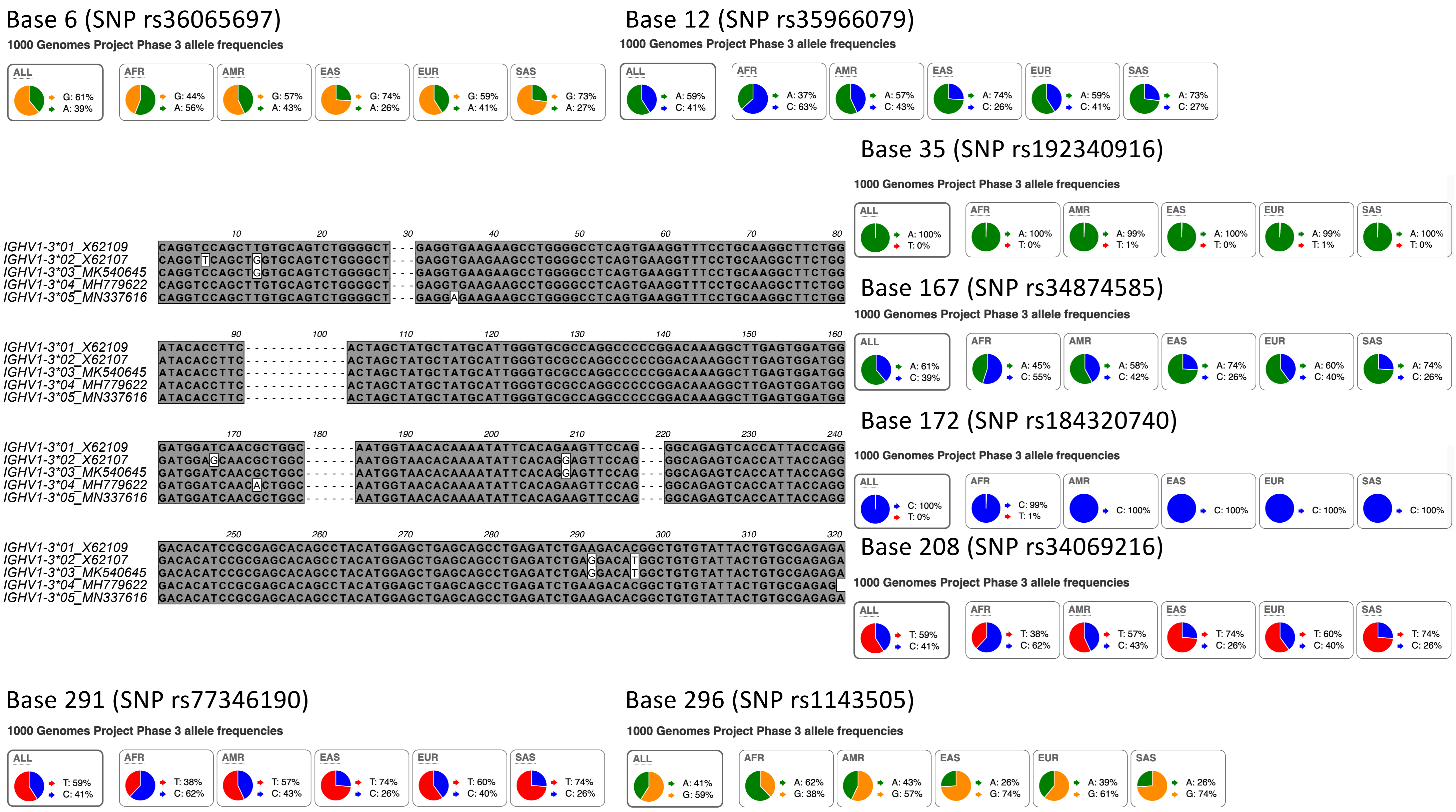

Base 6 (SNP rs36065697)
1000 Genomes Project Phase 3 allele frequencies
ALL G: 61% A: 39%
AFR G: 44% A: 56%
AMR G: 57% A: 43%
EAS G: 74% A: 26%
EUR G: 59% A: 41%
SAS G: 73% A: 27%
Base 12 (SNP rs35966079)
1000 Genomes Project Phase 3 allele frequencies
ALL A: 59% C: 41%
AFR A: 37% C: 63%
AMR A: 57% C: 43%
EAS A: 74% C: 26%
EUR A: 59% C: 41%
SAS A: 73% C: 27%
Base 35 (SNP rs192340916)
1000 Genomes Project Phase 3 allele frequencies
ALL A: 100% T: 0%
AFR A: 100% T: 0%
AMR A: 99% T: 1%
EAS A: 100% T: 0%
EUR A: 99% T: 1%
SAS A: 100% T: 0%
10 20 30 40 50 60 70 80
IGHV1-3*01_X62109 CAGGTCCAGCTTGTGCAGTCTGGGGCT---GAGGTGAAGAAGCCTGGGGCCTCAGTGAAGGTTTCCTGCAAGGCTTCTGG
IGHV1-3*02_X62107 CAGGTTCAGCTGGTGCAGTCTGGGGCT---GAGGTGAAGAAGCCTGGGGCCTCAGTGAAGGTTTCCTGCAAGGCTTCTGG
IGHV1-3*03_MK540645 CAGGTCCAGCTGGTGCAGTCTGGGGCT---GAGGTGAAGAAGCCTGGGGCCTCAGTGAAGGTTTCCTGCAAGGCTTCTGG
IGHV1-3*04_MH779622 CAGGTCCAGCTTGTGCAGTCTGGGGCT---GAGGTGAAGAAGCCTGGGGCCTCAGTGAAGGTTTCCTGCAAGGCTTCTGG
IGHV1-3*05_MN337616 CAGGTCCAGCTTGTGCAGTCTGGGGCT---GAGGAGAAGAAGCCTGGGGCCTCAGTGAAGGTTTCCTGCAAGGCTTCTGG
90 100 110 120 130 140 150 160
IGHV1-3*01_X62109 ATACACCTTC------------ACTAGCTATGCTATGCATTGGGTGCGCCAGGCCCCCGGACAAAGGCTTGAGTGGATGG
IGHV1-3*02_X62107 ATACACCTTC------------ACTAGCTATGCTATGCATTGGGTGCGCCAGGCCCCCGGACAAAGGCTTGAGTGGATGG
IGHV1-3*03_MK540645 ATACACCTTC------------ACTAGCTATGCTATGCATTGGGTGCGCCAGGCCCCCGGACAAAGGCTTGAGTGGATGG
IGHV1-3*04_MH779622 ATACACCTTC------------ACTAGCTATGCTATGCATTGGGTGCGCCAGGCCCCCGGACAAAGGCTTGAGTGGATGG
IGHV1-3*05_MN337616 ATACACCTTC------------ACTAGCTATGCTATGCATTGGGTGCGCCAGGCCCCCGGACAAAGGCTTGAGTGGATGG
170 180 190 200 210 220 230 240
IGHV1-3*01_X62109 GATGGATCAACGCTGGC------AATGGTAACACAAAATATTCACAGAAGTTCCAG---GGCAGAGTCACCATTACCAGG
IGHV1-3*02_X62107 GATGGAGCAACGCTGGC------AATGGTAACACAAAATATTCACAGGAGTTCCAG---GGCAGAGTCACCATTACCAGG
IGHV1-3*03_MK540645 GATGGATCAACGCTGGC------AATGGTAACACAAAATATTCACAGGAGTTCCAG---GGCAGAGTCACCATTACCAGG
IGHV1-3*04_MH779622 GATGGATCAACACTGGC------AATGGTAACACAAAATATTCACAGAAGTTCCAG---GGCAGAGTCACCATTACCAGG
IGHV1-3*05_MN337616 GATGGATCAACGCTGGC------AATGGTAACACAAAATATTCACAGAAGTTCCAG---GGCAGAGTCACCATTACCAGG
250 260 270 280 290 300 310 320
IGHV1-3*01_X62109 GACACATCCGCGAGCACAGCCTACATGGAGCTGAGCAGCCTGAGATCTGAAGACACGGCTGTGTATTACTGTGCGAGAGA
IGHV1-3*02_X62107 GACACATCCGCGAGCACAGCCTACATGGAGCTGAGCAGCCTGAGATCTGAGGACATGGCTGTGTATTACTGTGCGAGAGA
IGHV1-3*03_MK540645 GACACATCCGCGAGCACAGCCTACATGGAGCTGAGCAGCCTGAGATCTGAGGACATGGCTGTGTATTACTGTGCGAGAGA
IGHV1-3*04_MH779622 GACACATCCGCGAGCACAGCCTACATGGAGCTGAGCAGCCTGAGATCTGAAGACACGGCTGTGTATTACTGTGCGAGAG
IGHV1-3*05_MN337616 GACACATCCGCGAGCACAGCCTACATGGAGCTGAGCAGCCTGAGATCTGAAGACACGGCTGTGTATTACTGTGCGAGAGA
Base 167 (SNP rs34874585)
1000 Genomes Project Phase 3 allele frequencies
ALL A: 61% C: 39%
AFR A: 45% C: 55%
AMR A: 58% C: 42%
EAS A: 74% C: 26%
EUR A: 60% C: 40%
SAS A: 74% C: 26%
Base 172 (SNP rs184320740)
1000 Genomes Project Phase 3 allele frequencies
ALL C: 100% T: 0%
AFR C: 99% T: 1%
AMR C: 100%
EAS C: 100%
EUR C: 100%
SAS C: 100%
Base 208 (SNP rs34069216)
1000 Genomes Project Phase 3 allele frequencies
ALL T: 59% C: 41%
AFR T: 38% C: 62%
AMR T: 57% C: 43%
EAS T: 74% C: 26%
EUR T: 60% C: 40%
SAS T: 74% C: 26%
Base 291 (SNP rs77346190)
1000 Genomes Project Phase 3 allele frequencies
ALL T: 59% C: 41%
AFR T: 38% C: 62%
AMR T: 57% C: 43%
EAS T: 74% C: 26%
EUR T: 60% C: 40%
SAS T: 74% C: 26%
Base 296 (SNP rs1143505)
1000 Genomes Project Phase 3 allele frequencies
ALL A: 41% G: 59%
AFR A: 62% G: 38%
AMR A: 43% G: 57%
EAS A: 26% G: 74%
EUR A: 39% G: 61%
SAS A: 26% G: 74%
