# Supplementary material for: Poorly Expressed Alleles of Several Human Immunoglobulin Heavy Chain Variable Genes are Common in the Human Population
Source: Front Immunol. 2021 Feb 24;11:603980. doi: 10.3389/fimmu.2020.603980 (PMC7943739; doi:10.3389/fimmu.2020.603980)
Supplement: Supplementary Figure 5 — Allelic variants of IGHV4-4 as defined by IMGT are illustrated. Variability of some of the positions of these genes in samples obtained in different geographical locations as illustrated by the ENSEMBL browser (release 101, August 2020) (21) is shown. Analysis of this gene is complicated by extensive similarity with alleles of IGHV4-59 and IGHV4-61, alleles of which are also shown. A few of the positions of IGHV4-4 that display frequencies of variation >1% in all populations in the 1000 Genomes Project are shown. Note that variants at bases 46 and 308 [IMGT numbering nomenclature (20)], indicative of the IGHV4-4*01 allele are present at about 3-4% in European populations. All sequence variants of the illustrations of SNPs are indicated as seen in the reversed strand, hence they are complementary to the base of the coding strand. [file Image_5.pdf]

Supplementary Figure 5. Allelic variants of IGHV4-4 as defined by IMGT are illustrated. Variability of some of the positions of these genes in samples obtained in different geographical locations as illustrated by the ENSEMBL browser (release 101, August 2020) (Yates et al., 2020) is shown. Analysis of this gene is complicated by extensive similarity with alleles of IGHV4-59 and IGHV4-61, alleles of which are also shown. A few of the positions of IGHV4-4 that display frequencies of variation >1% in all populations in the 1000 Genomes Project are shown. Note that variants at bases 46 and 308 (IMGT numbering nomenclature (Lefranc, 2011)), indicative of the IGHV4-4*01 allele are present at about 3-4% in European populations. All sequence variants of the illustrations of SNPs are indicated as seen in the reversed strand, hence they are complementary to the base of the coding strand.

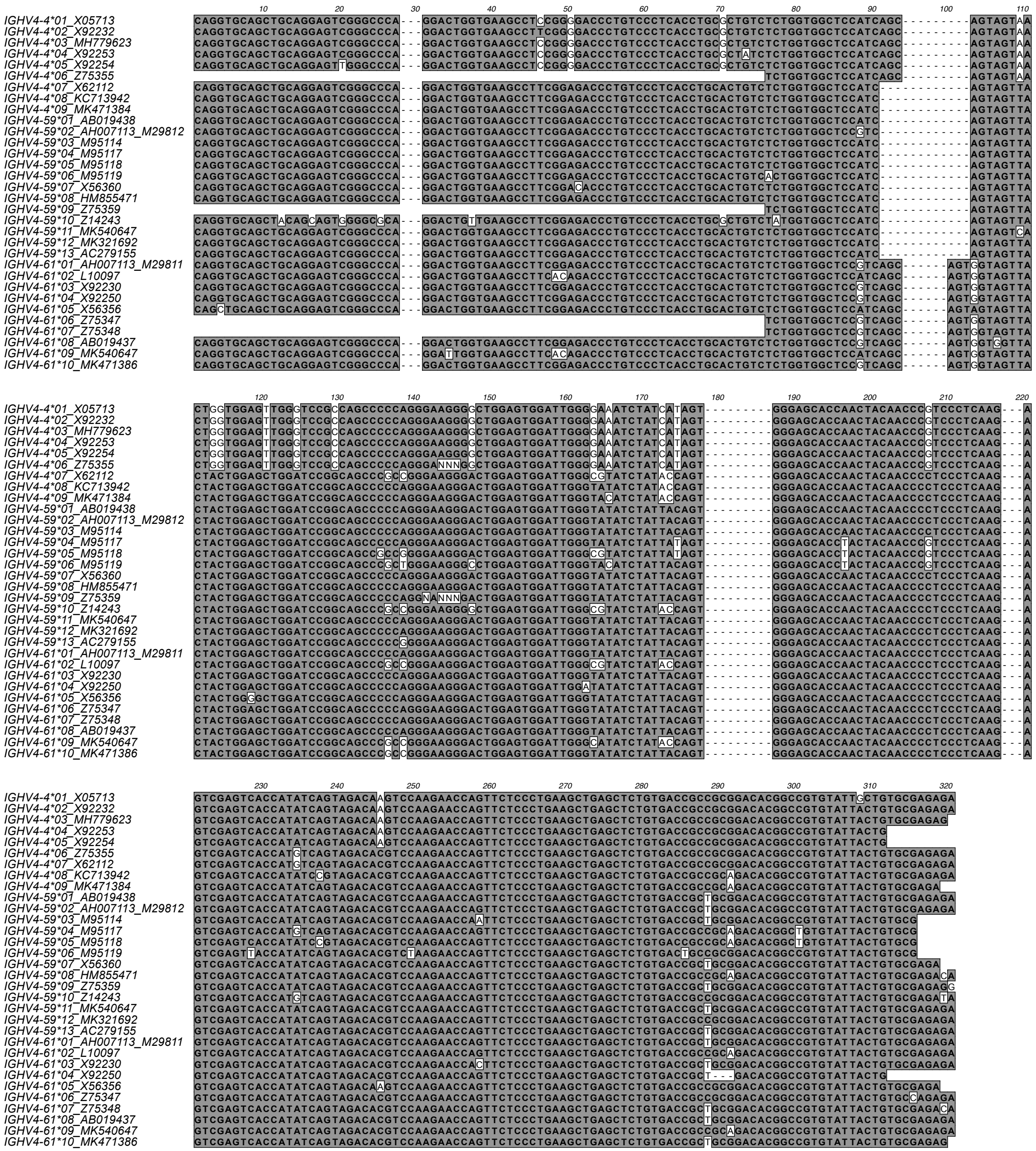

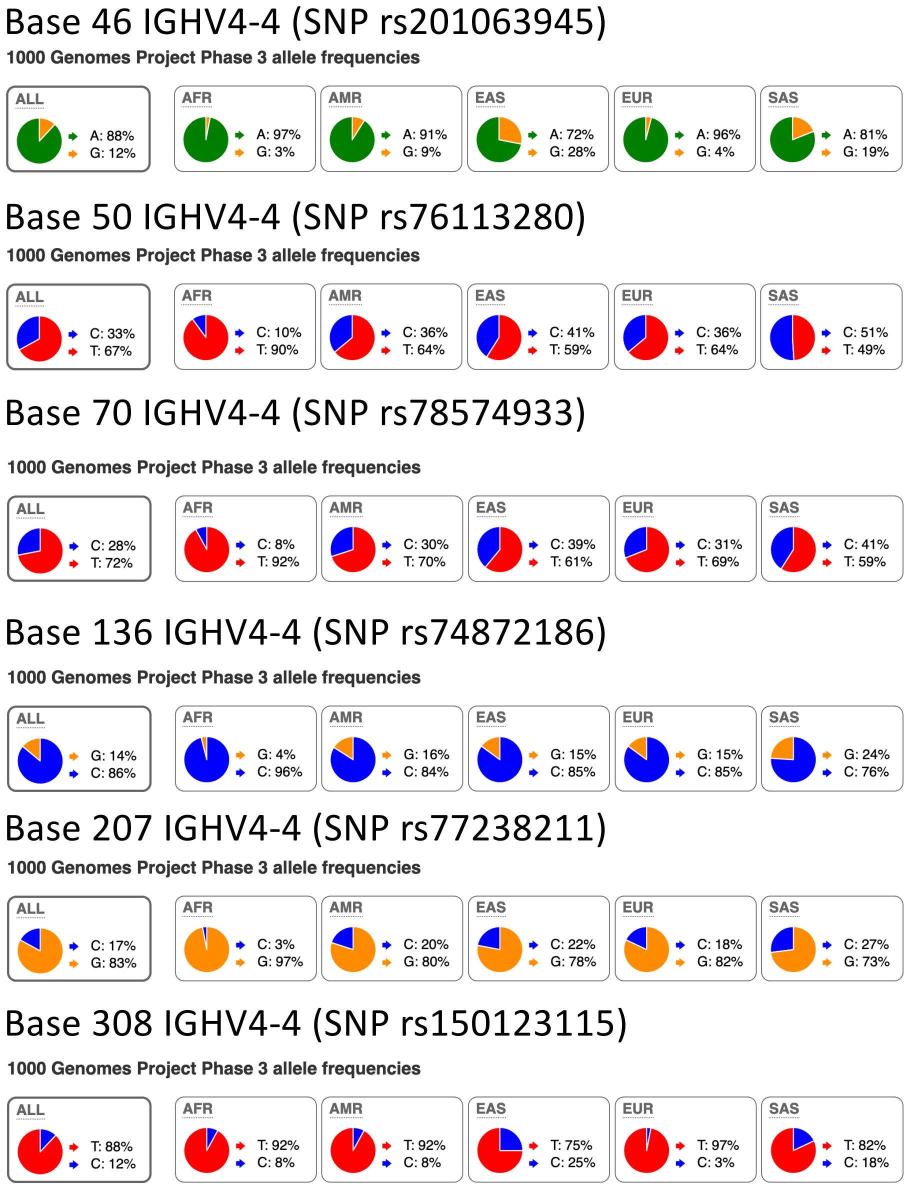

Base 46 IGHV4-4 (SNP rs201063945)
1000 Genomes Project Phase 3 allele frequencies
ALL A: 88% G: 12%
AFR A: 97% G: 3%
AMR A: 91% G: 9%
EAS A: 72% G: 28%
EUR A: 96% G: 4%
SAS A: 81% G: 19%
Base 50 IGHV4-4 (SNP rs76113280)
1000 Genomes Project Phase 3 allele frequencies
ALL C: 33% T: 67%
AFR C: 10% T: 90%
AMR C: 36% T: 64%
EAS C: 41% T: 59%
EUR C: 36% T: 64%
SAS C: 51% T: 49%
Base 70 IGHV4-4 (SNP rs78574933)
1000 Genomes Project Phase 3 allele frequencies
ALL C: 28% T: 72%
AFR C: 8% T: 92%
AMR C: 30% T: 70%
EAS C: 39% T: 61%
EUR C: 31% T: 69%
SAS C: 41% T: 59%
Base 136 IGHV4-4 (SNP rs74872186)
1000 Genomes Project Phase 3 allele frequencies
ALL G: 14% C: 86%
AFR G: 4% C: 96%
AMR G: 16% C: 84%
EAS G: 15% C: 85%
EUR G: 15% C: 85%
SAS G: 24% C: 76%
Base 207 IGHV4-4 (SNP rs77238211)
1000 Genomes Project Phase 3 allele frequencies
ALL C: 17% G: 83%
AFR C: 3% G: 97%
AMR C: 20% G: 80%
EAS C: 22% G: 78%
EUR C: 18% G: 82%
SAS C: 27% G: 73%
Base 308 IGHV4-4 (SNP rs150123115)
1000 Genomes Project Phase 3 allele frequencies
ALL T: 88% C: 12%
AFR T: 92% C: 8%
AMR T: 92% C: 8%
EAS T: 75% C: 25%
EUR T: 97% C: 3%
SAS T: 82% C: 18%
