# Supplementary material for: Poorly Expressed Alleles of Several Human Immunoglobulin Heavy Chain Variable Genes are Common in the Human Population
Source: Front Immunol. 2021 Feb 24;11:603980. doi: 10.3389/fimmu.2020.603980 (PMC7943739; doi:10.3389/fimmu.2020.603980)

Supplementary Figure 6. Allelic variants of IGHV7-4-1 as defined by IMGT are illustrated. Variability of some of the positions of these genes in samples obtained in different geographical locations as illustrated by the ENSEMBL browser (release 101, August 2020) (Yates et al., 2020) is shown. Sequence variation in base 274 (IMGT numbering nomenclature (Lefranc, 2011)) suggests that the base associated to IGHV7-4-1*01 is more common than the base associated to other alleles of this gene in most populations. All sequence variants of the illustrations of SNPs are indicated as seen in the reversed strand, hence they are complementary to the base of the coding strand.

```
                          10        20        30        40        50        60        70        80
IGHV7-4-1*01_IMGT000035   CAGGTGCAGCTGGTGCAATCTGGGTCT---GAGTTGAAGAAGCCTGGGGCCTCAGTGAAGGTTTCCTGCAAGGCTTCTGG
IGHV7-4-1*02_X62110       CAGGTGCAGCTGGTGCAATCTGGGTCT---GAGTTGAAGAAGCCTGGGGCCTCAGTGAAGGTTTCCTGCAAGGCTTCTGG
IGHV7-4-1*03_X92290       CAGGTGCAGCTGGTGCAATCTGGGTCT---GAGTTGAAGAAGCCTGGGGCCTCAGTGAAGGTTTCCTGCAAGGCTTCTGG
IGHV7-4-1*04_HM855485     CAGGTGCAGCTGGTGCAATCTGGGTCT---GAGTTGAAGAAGCCTGGGGCCTCAGTGAAGGTTTCCTGCAAGGCTTCTGG
IGHV7-4-1*05_HM855361     CAGGTGCAGCTGGTGCAATCTGGGTCT---GAGTTGAAGAAGCCTGGGGCCTCAGTGAAGGTTTCCTGCAAGGCTTCTGG

                                   90       100       110       120       130       140       150       160
IGHV7-4-1*01_IMGT000035   ATACACCTTC------------ACTAGCTATGCTATGAATTGGGTGCGACAGGCCCCTGGACAAGGGCTTGAGTGGATGG
IGHV7-4-1*02_X62110       ATACACCTTC------------ACTAGCTATGCTATGAATTGGGTGCGACAGGCCCCTGGACAAGGGCTTGAGTGGATGG
IGHV7-4-1*03_X92290       ATACACCTTC------------ACTAGCTATGCTATGAATTGGGTGCGACAGGCCCCTGGACAAGGGCTTGAGTGGATGG
IGHV7-4-1*04_HM855485     ATACACCTTC------------ACTAGCTATGCTATGAATTGGGTGCGACAGGCCCCTGGACAAGGGCTTGAGTGGATGG
IGHV7-4-1*05_HM855361     ATACACCTTC------------ACTAGCTATGCTATGAATTGGGTGCGACAGGCCCCTGGACAAGGGCTTGAGTGGATGG

                                  170       180       190       200       210       220       230       240
IGHV7-4-1*01_IMGT000035   GATGGATCAACACCAAC------ACTGGGAACCCAACGTATGCCCAGGGCTTCACA---GGACGGTTTGTCTTCTCCTTG
IGHV7-4-1*02_X62110       GATGGATCAACACCAAC------ACTGGGAACCCAACGTATGCCCAGGGCTTCACA---GGACGGTTTGTCTTCTCCTTG
IGHV7-4-1*03_X92290       GATGGATCAACACCAAC------ACTGGGAACCCAACGTATGCCCAGGGCTTCACA---GGACGGTTTGTCTTCTCCTTG
IGHV7-4-1*04_HM855485     GATGGATCAACACCAAC------ACTGGGAACCCAACGTATGCCCAGGGCTTCACA---GGACGGTTTGTCTTCTCCTTG
IGHV7-4-1*05_HM855361     GATGGATCAACACCAAC------ACTGGGAACCCAACGTATGCCCAGGGCTTCACA---GGACGGTTTGTCTTCTCCTTG

                                  250       260       270       280       290       300       310       320
IGHV7-4-1*01_IMGT000035   GACACCTCTGTCAGCACGGCATATCTGCAGATCTGCAGCCTAAAGGCTGAGGACACTGCCGTGTATTACTGTGCGAGAGA
IGHV7-4-1*02_X62110       GACACCTCTGTCAGCACGGCATATCTGCAGATCAGCAGCCTAAAGGCTGAGGACACTGCCGTGTATTACTGTGCGAGAGA
IGHV7-4-1*03_X92290       GACACCTCTGTCAGCACGGCATATCTGCAGATCAGCACGCTAAAGGCTGAGGACACTG
IGHV7-4-1*04_HM855485     GACACCTCTGTCAGCATGGCATATCTGCAGATCAGCAGCCTAAAGGCTGAGGACACTGCCGTGTATTACTGTGCGAGAGA
IGHV7-4-1*05_HM855361     GACACCTCTGTCAGCATGGCATATCTGCAGATCAGCAGCCTAAAGGCTGAGGACACTGCCGTGTGTTACTGTGCGAGAGA
```

Base 257 (SNP rs1172271390)

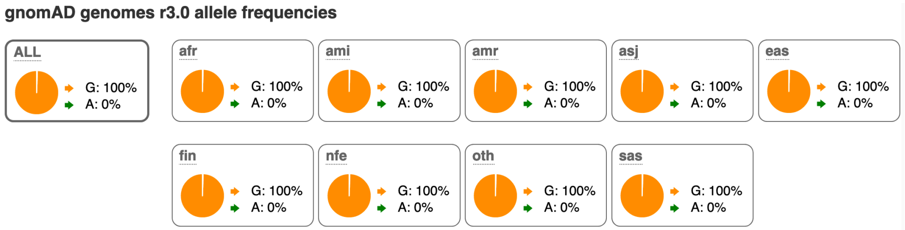

Base 274 (SNP rs1431854955)

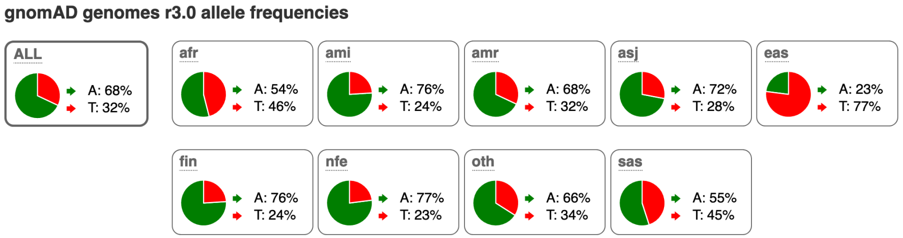

Supplement: Supplementary Figure 6 — Allelic variants of IGHV7-4-1 as defined by IMGT are illustrated. Variability of some of the positions of these genes in samples obtained in different geographical locations as illustrated by the ENSEMBL browser (release 101, August 2020) (21) is shown. Sequence variation in base 274 [IMGT numbering nomenclature (20)] suggests that the base associated to IGHV7-4-1*01 is more common than the base associated to other alleles of this gene in most populations. All sequence variants of the illustrations of SNPs are indicated as seen in the reversed strand, hence they are complementary to the base of the coding strand. [file Image_6.pdf]
