# Supplementary material for: Poorly Expressed Alleles of Several Human Immunoglobulin Heavy Chain Variable Genes are Common in the Human Population
Source: Front Immunol. 2021 Feb 24;11:603980. doi: 10.3389/fimmu.2020.603980 (PMC7943739; doi:10.3389/fimmu.2020.603980)
Supplement: Supplementary Figure 7 — High resolution structures of five antibodies with a heavy chain variable domain encoded by a gene derived from IGHV7-4-1. Heavy chain CDR3 is shown at the top of each structure in red. The side chain of residue 92 (in all cases a serine), located far from the antibody binding site is shown in green (carbon) and red (oxygen). Structures include PDB entries 4D9Q (A), 4EOW (B), 5CGY (C), 5ZMJ (D), and 6B5R (E). [file Image_7.pdf]

Supplementary Figure 7. High resolution structures of five antibodies with a heavy chain variable domain encoded by a gene derived from IGHV7-4-1. Heavy chain CDR3 is shown at the top of each structure in red. The side chain of residue 92 (in all cases a serine), located far from the antibody binding site is shown in green (carbon) and red (oxygen). Structures include PDB entries 4D9Q (A), 4EOW (B), 5CGY (C), 5ZMJ (D), and 6B5R (E).

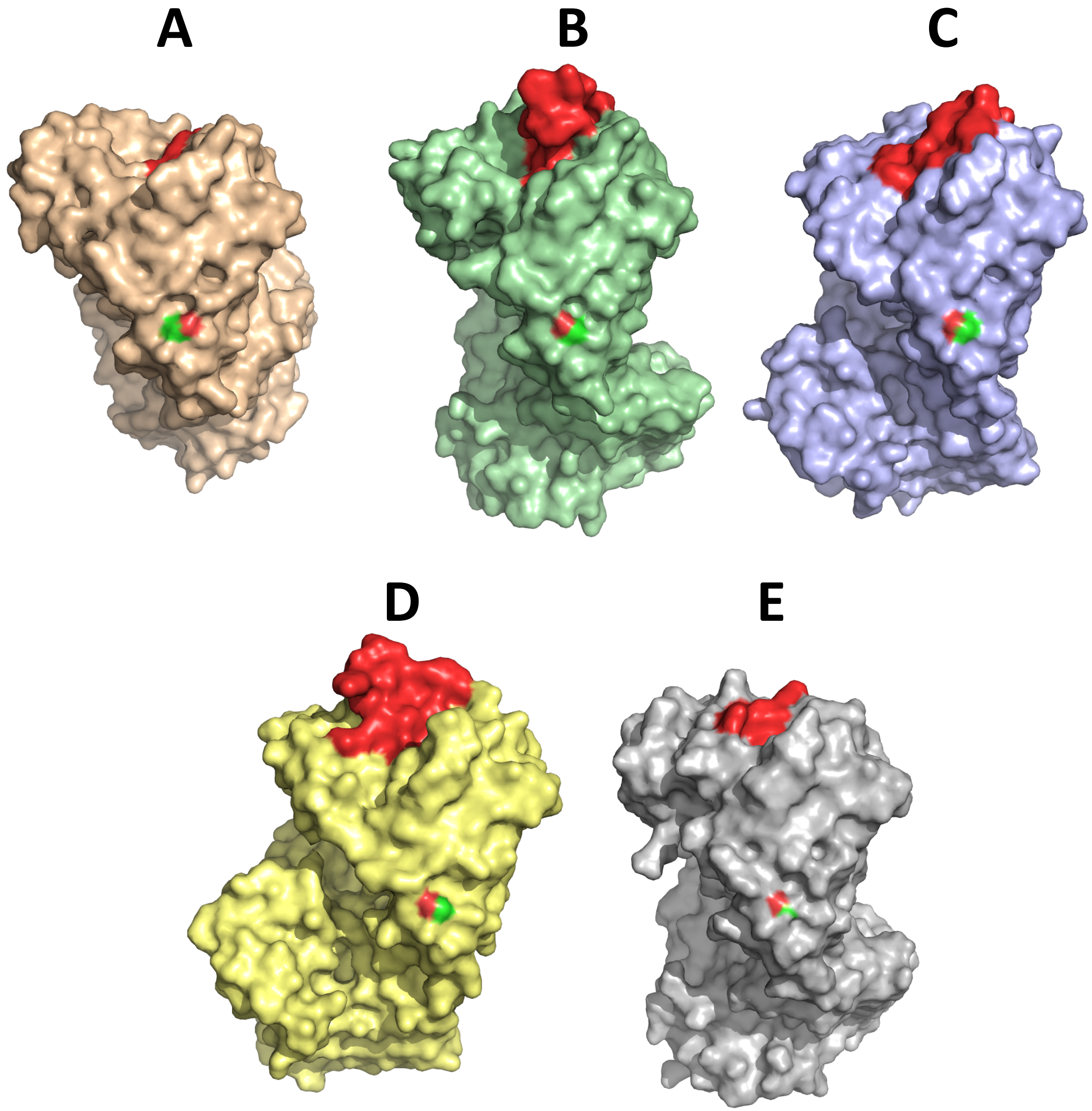
A
B
C
D
E
