# Supplementary material for: Poorly Expressed Alleles of Several Human Immunoglobulin Heavy Chain Variable Genes are Common in the Human Population
Source: Front Immunol. 2021 Feb 24;11:603980. doi: 10.3389/fimmu.2020.603980 (PMC7943739; doi:10.3389/fimmu.2020.603980)

Supplementary Figure 8. Translated sequences of productive IgA and IgG-encoding reads derived from NGS data sets of two subjects (donors 2 and 4) that both have IGHV7-4-1*01 but not IGHV7-4-1*02 in their genotype (Kirik et al., 2017b). The sequencing protocol (Levin et al., 2017) allowed for determination of the sequence from the end of framework 1 and extended into the first constant domain of the heavy chain. The sequences encoded by IGHV7-4-1*01 and IGHV7-4-1*02 are shown on top of the figure. Residue 92 is highlighted by an arrow.

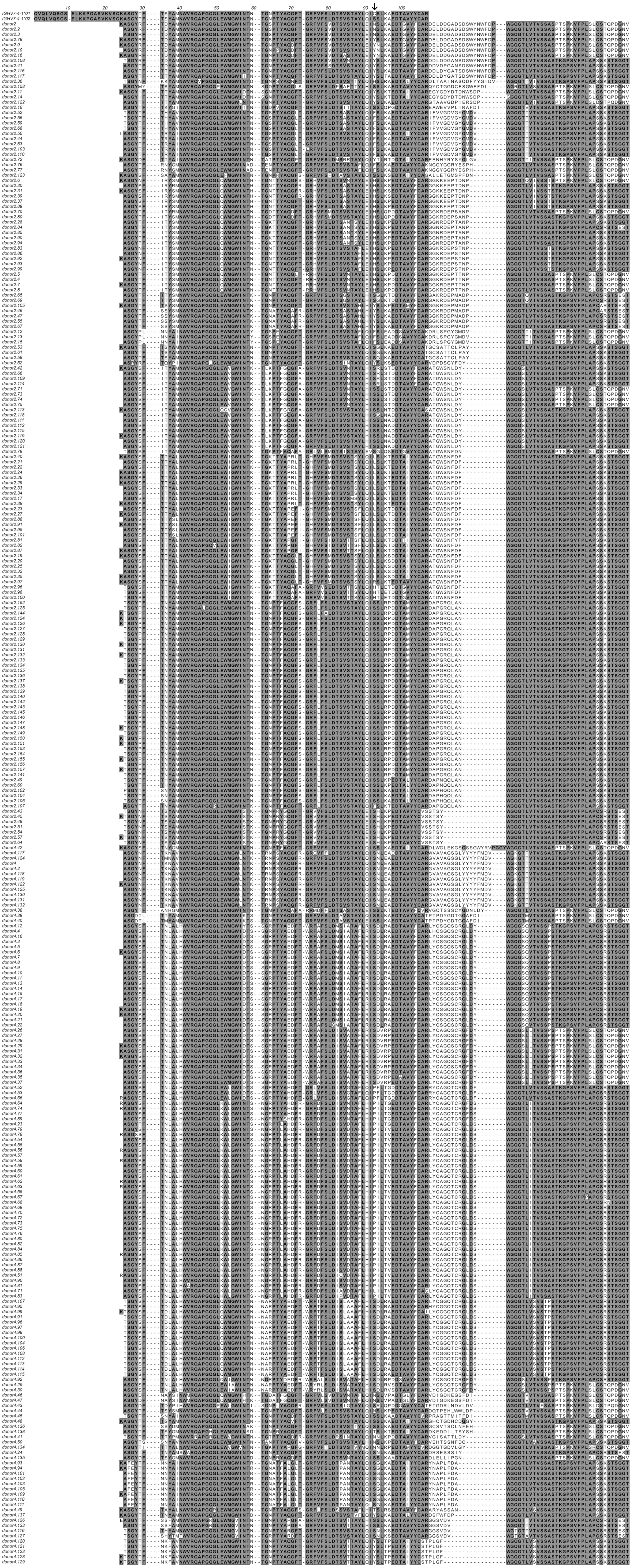

Supplement: Supplementary Figure 8 — Translated sequences of productive IgA and IgG-encoding reads derived from NGS data sets of two subjects (donors 2 and 4) that both have IGHV7-4-1*01 but not IGHV7-4-1*02 in their genotype (15). The sequencing protocol (19) allowed for determination of the sequence from the end of framework 1 and extended into the first constant domain of the heavy chain. The sequences encoded by IGHV7-4-1*01 and IGHV7-4-1*02 are shown on top of the figure. Residue 92 is highlighted by an arrow. [file Image_8.pdf]
