# Supplementary figures and images for: Poorly Expressed Alleles of Several Human Immunoglobulin Heavy Chain Variable Genes are Common in the Human Population
Source: Front Immunol. 2021 Feb 24;11:603980. doi: 10.3389/fimmu.2020.603980 (PMC7943739; doi:10.3389/fimmu.2020.603980)

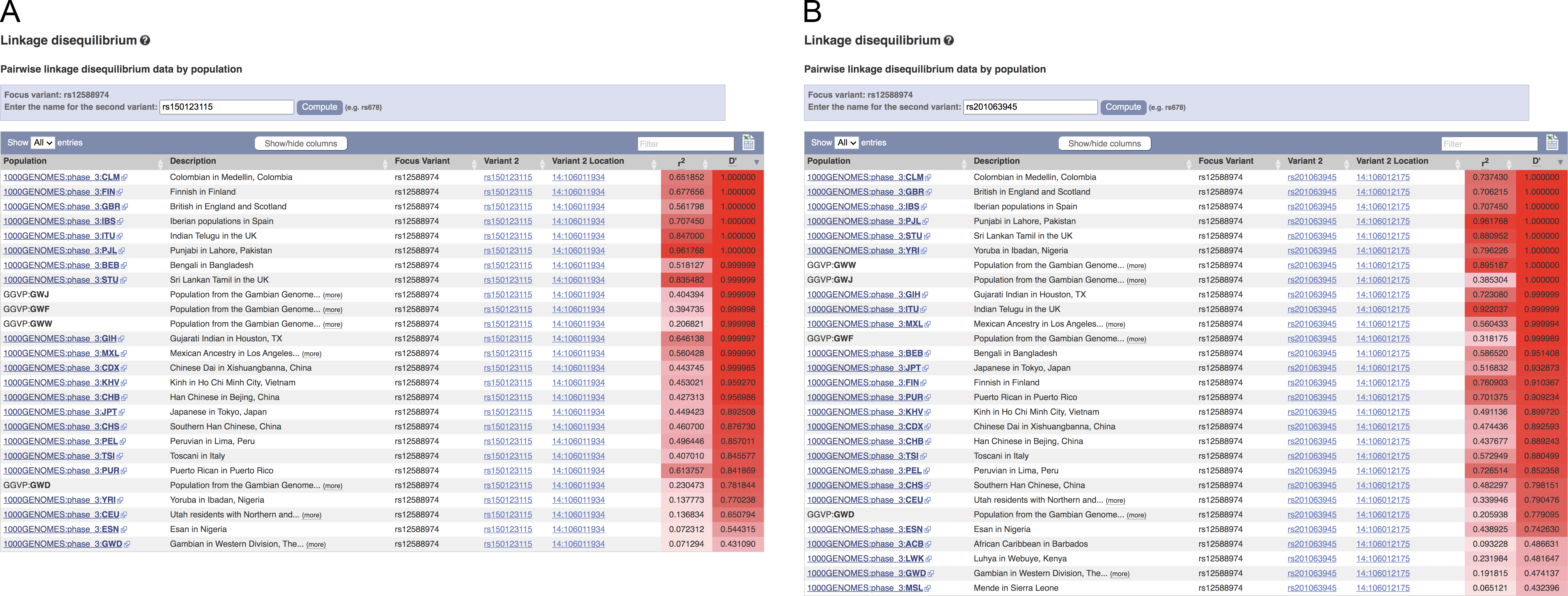

Supplement: Supplementary Figure 9 — Linkage equilibrium involving SNPs associated to IGHV1-2*05 and IGHV4-4*01 is identified in many populations [ENSEMBL browser (release 101, August 2020) (21)]. SNP rs12588974 (base 299 of IGHV1-2) separates IGHV1-2*05 from other commonly used alleles of the gene (IGHV1-2*02, IGHV1-2*04, IGHV1-2*06) ( Supplementary Figure 3 ) while SNPs rs150123115 (base 308 of IGHV4-4) (A) and rs201063945 (base 46 of IGHV4-4) (B) separate IGHV4-4*01 from other commonly used alleles of the gene (IGHV4-4*02 and IGHV4-4*07) ( Supplementary Figure 5 ). [file Image_9.tif]

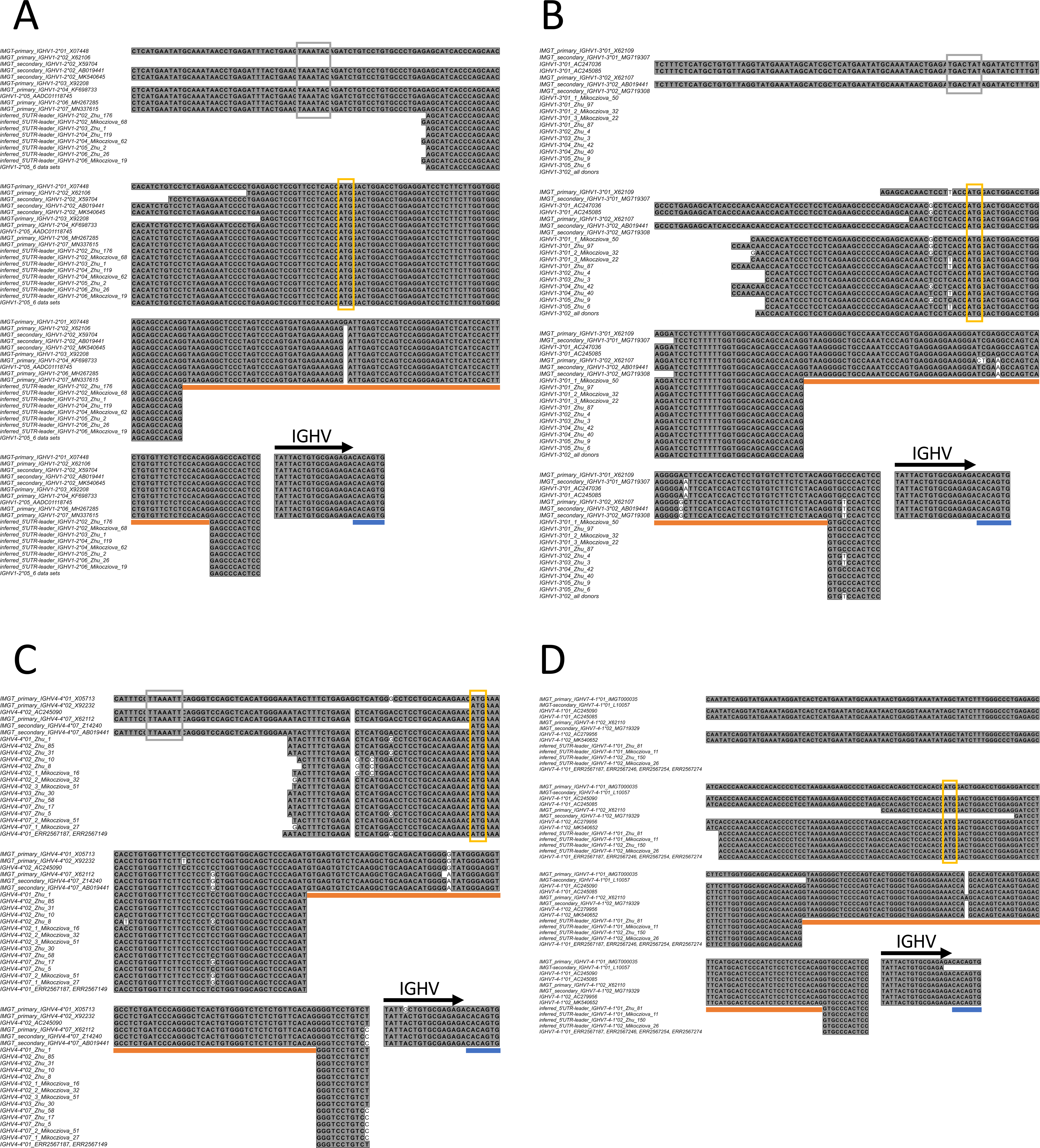

Supplement: Supplementary Figure 10 — Genomic germline gene sequences and inferred sequences representing the 5’- and 3’-end of major alleles and the poorly expressed alleles of IGHV1-2 (A), IGHV1-3 (B), IGHV4-4 (C), and IGHV7-4-1 (D). The TATA box (when identified in the IMGT database) and the ATG start codon are highlighted with a gray and yellow box, respectively. The intron sequence within the leader sequence is indicated by an orange line. Most of the sequence encoding the mature heavy chain variable domain (the end of which is indicated by an arrow) is not shown. Only bases starting from codon 102 and beyond, as well as the 3’-heptamer (indicated by a blue line) are shown. Genomic sequences were mostly retrieved from the IMGT website (http://www.imgt.org) but in a few cases directly from GenBank (https://www.ncbi.nlm.nih.gov/genbank/). The IMGT/LIGM-DB reference sequences is indicated as primary while other sequences derived from the IMGT website are indicated as secondary. Inferred sequences were obtained from publications by Zhu et al. (25) (of which only the more common variants are shown) and Mikocziova et al. (24). The number after these entries indicate the number of cases in which precisely this sequence had been inferred in these studies. At the bottom, the corresponding 5’UTR- and leader sequences of IGHV1-2*05, IGHV1-3*02, IGHV4-4*01, and IGHV7-4-1*01, as identified in data sets in the present study, are shown. [file Image_10.tif]
